# The effects of long-term physical activity interventions in communities: Scoping review in the Nordic countries

**DOI:** 10.1177/14034948211020599

**Published:** 2021-06-28

**Authors:** Elsi H. Haverinen, Hanna M. Elonheimo, Hanna K. Tolonen, Pekka J. Jousilahti, Heini J.C. Wennman

**Affiliations:** Department of Public Health Solutions, Finnish Institute for Health and Welfare, Helsinki, Finland

**Keywords:** Physical activity, fitness, intervention, community, behaviour change

## Abstract

*Aims:* Physical activity (PA) is an important part of maintaining good overall health. Currently, the number of insufficiently physically active adults and children is alarmingly high worldwide. To tackle the challenge, several interventions have been conducted, however, current knowledge on intervention effectiveness is still inconclusive. This scoping review aimed to summarize the effects of long-term PA interventions across all age groups in the Nordic countries. *Methods:* A scoping review was conducted by including all age groups and interventions lasting more than 12 months. The aims of the interventions had to focus on increasing PA and/or fitness. The Behaviour Change Wheel framework was used to describe components of the intervention functions. *Results:* Initially, 1937 studies were identified. Twelve intervention studies fulfilled the inclusion criteria and were included in the analysis. From the included studies, seven focused on children and/or their parents and five on working-age adult populations. Most of the studies built on theoretical backgrounds and included several behaviour change functions. A hindering factor for synthesis was variation in measurement methods: both subjective and objective outcome measures were reported. Among all age groups, intervention effects on PA were modest. ***Conclusions:* There was no clear evidence of increased PA or fitness from long-term interventions in communities. However, even small improvements in PA are important for increasing PA at a population level and enhancing public health. More research is required for evidence-based community and public health planning**.

## Background and aims

The prevalence of physical activity (PA) in children, adolescents and adults is alarmingly low worldwide. To tackle this challenge, increasing PA among different populations is a key target in the preventive work against noncommunicable diseases [1,2]. In this review, PA consists of both leisure time pursuits and PA in work-, school- or community-based settings, in households and in relation to transport [3,4]. In 2018, The World Health Organization (WHO) established a global action plan aiming to reduce the prevalence of insufficiently active adults and adolescents worldwide by 15% by 2030 [5]. The action plan is based on four main areas: society, environment, people and systems. To achieve the goal of increasing PA, action-enhancing work across all these areas is required.

In the Nordic countries (Finland, Sweden, Norway, Denmark, Iceland) the number of physically active people is among the highest in Europe [6], however PA levels are still far from the WHO’s PA recommendations [7,8]. According to the Nordic Monitoring Study [9], which aimed to assess the amount of PA in the Nordic countries between the years 2011 and 2014, approximately 41% of 7 to 12 year-olds and 66% of 18 to 65 year-olds were adequately physically active based on the WHO recommendations of 150 min of moderate-intensity PA per week for adults and 60 min/day for children and adolescents [8,10]. Based on each country’s own data [11–14] the estimated prevalence of adults (16–64 years) reaching sufficient PA levels were highest in Iceland (73%) and Denmark (72%). The lowest prevalence rate was in Finland (56%), but this is not directly comparable because it included adults 30–64 years of age. In older adults (⩾65 years) the highest rates were in Iceland and Denmark (68%) and the lowest rate was in Finland (36%). In children aged 10 to 11 the figure was highest in Finland (45%) and lowest in Denmark (16%); in adolescents aged 14 to 15, the highest figure was in Iceland (20%) and the lowest in Denmark (11 %) [11–14].

It is widely acknowledged that even small changes in PA can bring health benefits, especially among those with initially low PA levels [1,15]. Therefore, even a small increase in PA among the least active groups could increase overall population health. Not only does PA per se benefit health, but it is also an important contributor to the development of cardiorespiratory fitness (CRF) and thereby influences cardiovascular (CV) outcomes several years later [16]. Better CRF is a particularly strong predictor of cardiovascular health [17] and the current levels of CRF among children and adults are worrisome [18,19]. Another related concern for Nordic public health is the high sedentary time reported in different populations [20]. The prevailing sedentary lifestyle of children and adults calls for action, as longitudinal and time trend data suggest no change or even a slight increase in time spent being sedentary [21,22].

Health intervention programmes can be designed as multilevel interventions, or focus solely on individual, community or societal levels [23]. In this review, we describe interventions taking place in communities, which were defined by McQueen et al. [24] as a group of diverse people participating in an act or intervention, linked through social ties, sharing common perspectives and/or participating in joint actions in geographical locations or settings. It is widely recognized that support from family members, school environment, workplaces, supportive social services and communities are potential resources that can be harnessed to promote health and healthy lifestyle behaviours [25–27]. Knowing what features lay the groundwork for successful interventions, therefore, enables better replication of effective approaches in future interventions. The features of PA interventions in this review have been summarized on a variety of levels, ranging from information about the duration and delivery format of the intervention, to the recording of underlying behaviour change functions [28,29].

As opposed to individually tailored interventions, the community PA approach has the advantage of reaching out to a large number of people in their natural environment [30]. However, a limitation of this approach is the possibility of not sufficiently reaching those who are least active [30,31]. To date, evidence of the effectiveness of community interventions increasing population PA is promising, but nonetheless inconsistent and limited [31,32].

The Behaviour Change Wheel (BCW) framework introduced by Michie et al. [33], was chosen as the model to be used in this review, as it provides an easy-to-access method of characterizing and planning health behaviour interventions and functions at different levels. The model considers individual characteristics (sources of behaviour), intervention methods (intervention functions) and larger societal characteristics (policy categories), which can be significant contributors to the success or failure of an intervention. At the centre of the framework lies the behaviour system, or sources that generate behaviour: capability, opportunity and motivation. Around the centre there are nine intervention functions and seven policy categories that build on this. The intervention functions target the deficits in capability, opportunity and motivation and interact with them, while the policy categories enable the interventions to take place and interact at the function level. The BCW framework derives from earlier applied classifications of behaviour intervention functions in order to build an overarching model of behaviour and link the interventions to behavioural targets. This model is used in the article to describe the similarities and differences in intervention actions and functions rather than to focus on individual behaviour characteristics.

Taken together, there remains a need to broaden understanding of how to increase people’s PA, and identify which intervention methods may prove most beneficial for behaviour change. Examination of long-term interventions in the Nordic communities among diverse age-groups will also make possible the further examination of current knowledge, and identification of the strengths and limitations of intervention studies and their methodologies. The aim of this scoping review was to synthesize features of community PA interventions in the Nordic countries, focusing on studies that aim to increase PA or CRF in a large group of people over the long term. Furthermore, the goal was to identify current gaps in community PA interventions to inform and advance future intervention planning and reporting.

## Methods

This study was conducted as a scoping review. The framework of Levac et al. [34], based on Arksey and O’Malley [35] was followed during the process.

### Identifying the research questions

The research team agreed on the research questions and modified the scope of the review during the process. Two research questions were set: 1) What is known about long-term PA interventions taking place in communities in the Nordic countries? and 2) What characteristics in an intervention support/hinder successful outcomes in regard to PA?

### Identifying relevant studies

A literature search was conducted from eight electronic databases (Medline, Cinahl, PsycInfo, Cochrane Database of Systematic Reviews, Cochrane Central Database of Controlled Trials, Medic, Melinda and Finna) in June 2019. The search terms are included in the Supplemental Material and Table I presents the inclusion criteria.

**Table I.** Inclusion criteria.

Randomized controlled trials with over 60 participants
Cohort studies with over 200 participants
Included a PA intervention
The intervention was described in adequate detail
All age groups
Had to report a PA or fitness outcome
Duration (of intervention only or intervention + follow-up) at least 12 months
Dropout less than 30%

PA: physical activity.

### Study selection

**Figure 1.**
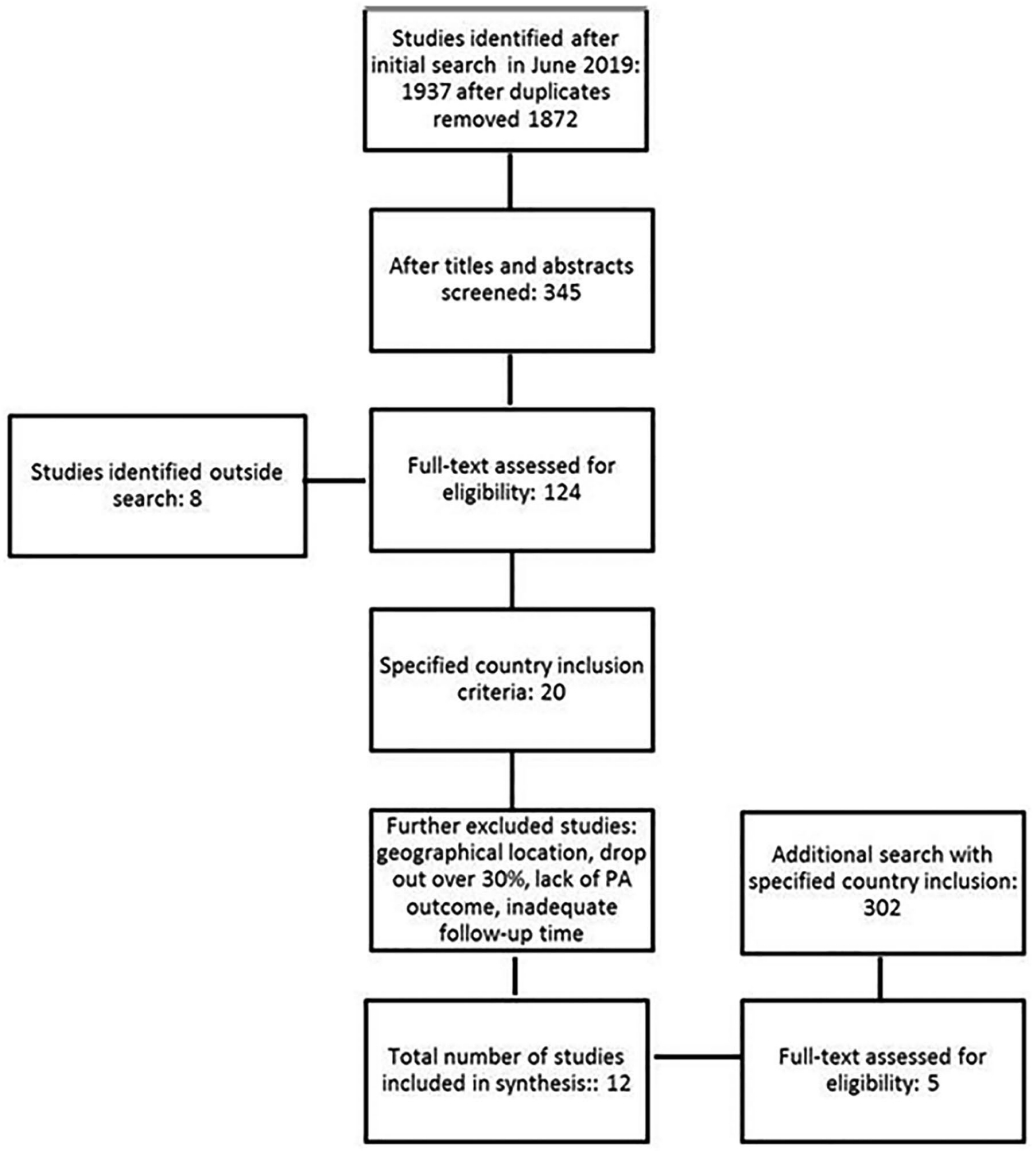
Flowchart of study selection.

In the initial literature search, 1937 studies were identified, and the results were exported to RefWorks database. Authors (HW, HE, EH) participated in the scanning of titles and abstracts, which identified 345 studies for further examination. At this stage the country inclusion was narrowed to include only the Nordic countries, which all have a so-called Nordic model comprising similar economic and social structures, as well as cultural practices between the countries [36]. This means that these countries support a market economy and universal welfare state finances with high taxes. This allows a high degree of income redistribution, the availability of free or almost free healthcare, free education, and children attending municipal day care, that is, equal opportunities for all. The limitation to the countries included will help to produce a more comparable analysis of what intervention functions, methods or tools may be successful [36]. Due to the change in the country criterion a complementary search was conducted entailing Nordic settings, providing 302 additional studies, from which five studies were assessed for eligibility. Outside this search, eight studies were included from references and other sources. The literature search was based on English peer-reviewed academic articles only. A flow diagram of the search strategy and accepted publications are presented in Figure 1.

### Charting the data

The selected studies (10 interventions, 12 studies) were charted according to the BCW framework. Each article was extracted for detailed information concerning participant demographics, intervention description, primary and secondary outcomes and theoretical background. Notably, the original articles varied in relation to the contexts the interventions were conducted in. The included studies were published between years 2009 and 2019. The interventions were conducted as randomized controlled trials. The results were charted according to age categories (Tables II and III).

**Table II.** Summary of family-based intervention [37] and school-based interventions [39–44]..

Authors	Intervention actions/ intervention age group	Theoretical background	Outcome(s) PA, devices and cut-off points	Outcome(s) others	Effect(s) (positive impact, null results, negative impact)	Intervention functions according to the BCW [33]
Laukkanen et al. [37]	Tailored counselling for parents to increase their children’s PA: 1) a lecture, 2) individual face-to-face counselling and goal-setting, 3) counselling by phone. 4–7 years (+ parents, see Pesola et al. [38]).	Social cognitive theory; theory of planned behaviour.	**Outcome:** Motor control tests (Körperkoordinationstest für Kinder, KTK): walking backwards, hopping for height, jumping sideways, moving sideways. Throwing and catching a ball (TCB) test. Accelerometer-assessed PA and sedentary time **Device:** triaxial X6-1A USB accelerometer; **Location:** waist; **Cut-off points:** 373 counts/15 s for light, 585 counts/15 s for moderate and 881 counts/15 s for vigorous PA. **Additional information:** proportional values of time spent at different PA intensities of sedentary, light and moderate-to-vigorous PA (MVPA) were calculated in relation to the total measurement time and by weighting weekdays by 5/7 and weekend days by 2/7.	Weight, height, BMI.	A significant decrease of MVPA in the intervention group when compared with the control group (mean difference −0.16% [−0.32 to 0.001], *p* = 0.033); **0** Nonsignificant difference in change between groups in sedentary time (mean difference 0.11% [−0.02 to 0.25], *p* = 0.106); **0** Nonsignificant difference in change between groups in light PA (mean difference −0.02% [−0.13 to 0.09], *p* = 0.285); **0** Nonsignificant difference in change between groups in TCB (*p* = 0.984); **0** Nonsignificant difference in change between groups in KTK score (mean difference −1.47 [−9.52 to 6.58], *p* = 0.737). **+** the intervention group had a steadier development of KTK score when the interaction with season was also taken into account (*p* for group*time*season = 0.008).	Education, persuasion, modelling.
Gråsten et al. [39]	Task-involving climate treatment: teacher training and task-involving climate support in physical education (PE) classes. Physical school environment treatment: development of the physical environment of the school and providing of equipment. 12–14 years.	Achievement goal theory; social ecological model.	**Outcome:** self-reported frequency of MVPA in a typical week and during the last 7 days **Method:** assessed with the Health Behavior in School-Aged Children Research Protocol – a scale consisting of 2 items rated on an 8-point response scale (0 to 7 days of the week) **Additional information:** the mean scores of the 2 items were used as MVPA scores.	Perception of Success Questionnaire: ego- and task-orientation, achievement goal orientations; Motivational Climate in Physical Education Scale: motivational climate.	**+** intervention group reported 13.4% higher total MVPA in comparison to control group.	Education, environmental restructuring, enablement.
Kokkonen et al. [40]	Two PE teachers were trained in the creative physical education (CPE) method, who then provided training to the remaining teachers in workshops and hands-on practise sessions. CPE included PA classes, and other tasks such as team work exercises and training materials supporting the approach. 10–12 years.	CPE model-based intervention; self- determination therapy; achievement goal theory.	**Outcome:** leisure time PA and overall motivation to PA; **Method:** questionnaire-based scale (the Sport Motivation Scale); self-reported frequency of MVPA. **Additional information:** MVPA in a typical week and during the last 7 days summed to represent overall PA (the Health Behaviour in School-aged Children Research Protocol).	Sport Motivation Scale: task- and ego-support; Motivational Climate in Physical Education Scale: motivational climate in PE.	**0** No significant intervention effect on overall MVPA (*F*[2.306] = 1.55, *p* = 0.213, *n*^2^ <= 0.01).	Training, education, modelling.
**a**: Christiansen et al. [41] **b**: Christiansen et al. [42]	Improvements in the physical and organizational environment and educational activities; improvement of outdoor environment; mandatory outdoor recess; safe cycling routes near school; free access to schools’ sports halls; teachers as recess kick-starters. 11–14 years.	Social ecological framework.	**a:** **Outcome:** shuttle run (20 m, Andersen test) and handgrip tests; **Device:** a digital TAKEI-dynamometer for a handgrip test. **b:** **Outcome:** accelerometer-assessed MVPA; **Device:** Actigraph GT3X accelerometer; **Location:** waist; **Cut-off points:** daily PA was defined by minutes of MVPA using the Evenson activity cut points (MVPA 2296 cpm). **Additional information:** activity for all 24 h was included.	**a:** Waist circumference. **b:** Questionnaire-based schoolyard index.	**a:** **0** No significant difference between intervention and control groups in shuttle run results at follow-up (mean difference 6 m; 95% confidence interval (CI) −20 to 31, *p* = 0.43). Significant difference in handgrip between intervention and control groups at follow-up (mean difference −1.1kg; 95%CI −2.2 to −0.0, *p* = 0.05). **b:** Total PA (MVPA min/day) higher in control schools than intervention schools at follow-up (mean MVPA 44. 8 min/day in intervention schools vs. 49.9 min/day in control schools, *p* = 0.003 between groups). **0** Recess PA at follow-up 714 cpm in intervention schools and 642 cpm in control schools (*p* = 0.01 between groups), but the difference had already been observed at baseline.	Environmental restructuring, enablement, education, restriction.
Haapala et al. [43]	Schools had the possibility to tailor the intervention according to their needs; increased number of recesses, improvement of outdoor environment, joint PA training, procurement of bicycles. Support to schools from the programme in form of national seminars, program webpages and local mentors 7-15 years.	Social ecological model is mentioned.	**Outcome:** accelerometer-assessed MVPA and sedentary time during school day and outside school hours; **Device:** ActiGraph accelerometer (GT1M and GT3X); **Location:** hip; **Cut-off points:** applied cut-off points were based on Evenson et al. (2008) to calculate ST (<100 cpm) and MVPA (>2295 cpm). Spurious data avoided by setting a 20,000 cpm upper limit (Heil et al. 2012). **Additional data sources:** diary to monitor school and sleeping hours and types of activities that the accelerometer did not measure (such as cycling, strength training and swimming).	N/A	**Primary grade schools:** **+** increase of school day MVPA (*b* = 0.27 [SE 0.10], *p* = 0.010) **+** decrease of school day sedentary time greater (*b* −0.52 [SE 0.20], *p* = 0.008) in primary schools undertaking the intervention as compared to control schools. **0** Nonsignificant difference in change between intervention and control schools in leisure time MVPA l (min/day) (*b* = 0.13, [0.99], *p* = 0.894). **0** Nonsignificant difference in change between intervention and control schools in leisure time sedentary time (min/h) (*b* = 0.04, [SE 0.6], *p* = 0.879). **0** Nonsignificant difference in change between intervention and control schools in total MVPA (min/day) (*b* = 1.33, [SE 0.26], *p* = 0.250). **0** Nonsignificant difference in change between intervention and control schools in total sedentary time (min/h) (*b* = −0.19, [0.35], *p* = 0.584). **Lower secondary-grade schools:** **0** Nonsignificant difference in change between intervention and control schools in school day MVPA (min/h) (*b* = 0.06, [SE 0.11], *p* = 0.561). **0** Nonsignificant difference in change between intervention and control schools in school day SED (min/h) (*b* = −0.48, [0.26], *p* = 0.063). **0** Nonsignificant difference in change between intervention and control schools in leisure time MVPA (min/day) (*b* = −1.31, [1.48], *p* = 0.376). **0** Nonsignificant difference in change between intervention and control schools in leisure time SED (min/h) (*b* = 0.10, [SE 0.27], *p* = 0.709). **0** Nonsignificant difference in change between intervention and control schools in total daily MVPA (min/day) (*b* = −0.65, [SE 1.68], *p* = 0.698). **0** Nonsignificant difference in change between intervention and control schools in total SED (min/h) (*b* = 0.33, [SE 0.224], *p* = 0.162).	Environmental restructuring, education, persuasion, enablement.
Rexen et al. [44]	Extra PE lessons 270 min/week. PE teachers and pedagogues participated in a course of 40 lessons. A handbook containing inspiration for the content of PE lessons, practical suggestions, and exercises. 6–10 years.	No theoretical background.	**Outcome:** composite *z*-score of following six fitness tests including five motor performance tests and one aerobic performance test: shuttle run, handgrip, vertical jump, KTK, (walking backwards, hopping for height, jumping sideways, moving sideways), precision throw, the Andersen test (20 m shuttle run).	N/A	**Over 2.5 years** **+** significant difference in change between intervention and control groups in fitness composite *z*-score in Cohort 4: difference 1.06 units (95%CI 0.48–1.65, *p* < 0.001). **+** borderline significant difference in change between intervention and control school in fitness composite score in Cohort 3: 0.52 units (95%CI −0.06 to 1.09, *p* = 0.078). **0** Nonsignificant difference in change between intervention and control schools in fitness composite score in Cohort 2: 0.10 (95%CI −0.47 to 0.66, *p* = 0.73). **0** Nonsignificant difference in change between intervention and control school in fitness composite score in Cohort 1: 0.46 (95%CI −0.12 to 1.04, *p* = 0.12). **0** Nonsignificant difference in change between intervention and control schools in fitness composite score in Cohort 0: −0.33 (95%CI −0.95 to 0.29, *p* = 0.29). **+** children in the lower 50 percentiles of baseline fitness level increased their development, 0.47 *z*-score units (95%CI 0.08–0.85, *p* = 0.018), compared with control school children.	Education, persuasion, enablement.

PA: physical activity; BMI: body mass index; BCW: Behaviour Change Wheel; HDL: high-density lipoprotein; ApoA1: Apolipoprotein A1; ApoB: Apolipoprotein B; LTPA: leisure-time physical activity.

**Table III.** Summary of the working-age interventions [38,45–48].

Authors	Intervention type/ intervention age group	Theoretical background	Outcome(s) PA, devices and cut-off points	Outcome(s) others	Effect(s) (positive impact, null results, negative impact)	Intervention functions according to the BCW [33]
Pesola et al. [38]	Tailored counselling for participants: 1) a lecture, 2) individual face-to-face counselling and contractual goal-setting, 3) counselling by phone. Office workers with young children: 28–53 years.	Motivational interview; theory of planned behaviour.	**Outcome:** accelerometer-assessed PA and sedentary time **Device:** alive-accelerometer **Location:** waistline **Cut-off points:** time spent sedentary (<100 counts/min), light (⩾ 100 < 2020 counts/min) and moderate-to-vigorous activity (⩾2020 counts/min) **Additional data sources:** a log sheet of working and bed times and activities without the accelerometer, and any abnormal behaviours possibly affecting the measurements.	Weight, BMI, arm fat mass, leg lean mass, total lean mass, total cholesterol, HDL, ApoA1, ApoB/ApoA1.	**0** Nonsignificant difference in change between groups in total sedentary time (min/16 h) at 12 months (mean difference −11.4 [−39.0, 16.4], *p* = 0.79). **0** Nonsignificant difference in change between groups in total light PA (min/16 h) at 12 months (mean difference 11.3 [−15.4, 38.1], *p* = 0.84). **0** Nonsignificant difference in change between groups in total moderate-to-vigorous PA (min/16 h) at 12 months (mean difference 0.4 [−6.5, 7.3], *p* = 0.39). **0** Nonsignificant difference in change between groups in total breaks per sedentary hour at 12 months (mean difference −0.3 [−1.2, 0.6], *p* = 0.88). **0** Nonsignificant difference in change between groups in worktime sedentary time (min/8 h) at 12 months (mean difference −0.1 [−22.0, 21.7], *p* = 0.95). **0** Nonsignificant difference in change between groups in worktime light PA (min/8 h) at 12 months (mean difference 0.1 [−20.6, 20.8], *p* = 0.98). **0** Nonsignificant difference in change between groups in worktime moderate-to-vigorous PA (min/8 h) at 12 months (mean difference 0.2 [−3.6, 4.0], *p* = 0.28). **0** Nonsignificant difference in change between groups in worktime breaks per sedentary hour at 12 months (mean difference −1.1 [−2.5, 0.4], *p* = 0.70). **0** Nonsignificant difference in change between groups in leisure time sedentary time (min/8 h) at 12 months (mean difference −7.9 [−24.0, 8.3], *p* = 0.26). **0** Nonsignificant difference in change between groups in leisure time light PA (min/8 h) at 12 months (mean difference 3.7 [−12, 19.4], *p* = 0.52). **0** Nonsignificant difference in change between groups in leisure time moderate-to-vigorous PA (min/8 h) at 12 months (mean difference 4.5 [−1.2, 10.2], *p* = 0.54). **0** Nonsignificant difference in change between groups in leisure time breaks per sedentary hour at 12 months (mean difference 0.6 [−0.6, 1.8], *p* = 0.53). **0** Nonsignificant difference in change between groups in weekend sedentary time (min/16 h) at 12 months (mean difference −25.6 [ −77.2, 26.1], *p* = 0.31). **0** Nonsignificant difference in change between groups in weekend light PA (min/16 h) at 12 months (mean difference 19.7 [−30.4, 69.8], *p* = 0.59). **0** Nonsignificant difference in change between groups in weekend moderate-to-vigorous PA (min/16 h) at 12 months (mean difference 7.4 [−9.9, 24.7], *p* = 0.22). **0** Nonsignificant difference in change between groups in weekend breaks per sedentary hour at 12 months (mean difference −0.2 [−2.0, 1.6], *p* = 0.16).	Education, persuasion, restriction.
**a:** Kettunen et al. [45] **b:** Kettunen et al.[46]	Two-day training camps 5 times during intervention and follow-up. 1–2 supervised exercise sessions in a group per month. 3–5 un-supervised exercise sessions per week. An individualized exercise programme based on the estimated oxygen uptake (VO₂max). Employees (20–60-years) from small- and medium-sized companies.	No theoretical background.	**a + b:** **Outcome:** cardiorespiratory fitness (VO₂max) **Device:** a sub-maximal cycle ergometer test with bicycle ergometer (Ergoline 800S) **Additional data sources:** self-reported frequency, intensity and duration of leisure time PA calculated into metabolic equivalent hours per week, personal exercise diary, lifestyle questionnaire. Exercise intensity measured with Polar heart rate microcomputers (Polar Electro).	**a**: Questionnaire-based Work Ability Index (WAI) **b:** Occupational Stress Questionnaire: Stress symptom index, and mental resource index.	**a + b:** **+** Difference in change in VO₂max between groups significant at 12- and 24 months (*p* = 0.008 and *p* = 0.015, respectively). VO₂max in intervention group at 12 months= +7% (*p* < 0.001), and after 24 months = +5% (*p* <= 0.01). VO₂max in control group at 12 months −3% and at 24 months not significantly different from baseline. **+** LTPA in intervention group at 12 months of 71% (*p* = 0.016). No significant change in LTPA in control group (+0.03%).	Training, education, persuasion.
Aittasalo et al. [47]	Preliminary worksite meeting with a researcher. Self-monitoring of PA with the pedometer and logbook. Monthly email message from Occupational Healthcare. Personal walking goals by adding daily number of steps during 5 days of a week. Office workers.	Health action process approach was used to formulate email messages.	**Outcome:** self-reported walking stairs, leisure time walking, walking at work, walking for transportation, total walking, time spent sitting during working day, time spent sitting during nonworking day **Method:** self-administered International Physical Activity Questionnaire, logbook.	RE-AIM (reach, effectiveness, adoption, implementation and maintenance), direct costs assessed with questionnaires, process evaluation and interviews.	**0** Nonsignificant difference in change between groups in weekly minutes of walking stairs at 12 months (geometric mean ratio (GMR) 1.18 [0.91–1.53]). **0** Nonsignificant difference in change between groups in the percentage of those walking stairs at 12 months (odds ratio (OR) 2.24 [0.94–5.31]). **0** Nonsignificant difference in change between groups in weekly minutes of leisure time walking at 12 months (GMR 1.21 [0.98–1.59]). **0** Nonsignificant difference in change between groups (%) walking for leisure at 12 months (OR 2.07 [0.99–4.34]). **0** Nonsignificant difference in change between groups in weekly minutes of walking for transportation (GMR 1.03 [0.77–1.39]). **0** Nonsignificant difference in change between groups (%) walking for transportation at 12 months (OR 1.57 [0.68–3.61]). **0** Nonsignificant difference in change between groups in weekly minutes of walking at work (GMR 1.13 [0.92–1.38]). **0** Nonsignificant difference in change between groups (%) walking at work at 12 months (OR 2.39 [0.15–37.3]). **0** Nonsignificant difference in change between groups in weekly minutes of total walking (GMR 1.25 [0.98–1.59]). **0** Nonsignificant difference in change between groups in minutes of sitting during working day at 12 months (mean difference −9 min [−56, 37]). **0** Nonsignificant difference in change between groups in minutes of sitting during nonworking day at 12 months (mean difference −9 min [−52, 33]).	Education, persuasion.
Hemmingsson et al. [48]	Behavioural counselling package: 1) three individual sessions with a physician, 2) two group counselling sessions, and 3) a new bicycle. Detailed PA prescriptions. Three meetings with a doctor to change PA behaviour.	Transtheoretical model; behaviour change theory.	**Outcome:** cycling (km/day) **Device:** cycling measured with a trip meter (Trelock FC 410), step count assessed with Yamax digiwalker SW-200 pedometer **Additional data sources:** compliance to intervention goals, frequency of self-reported commuting by cycling, walking more than 1 km, driving or using public transportation, assessed with diaries.	Waist circumference, body weight, saggital abdominal diameter.	**+** In intervention group 38.7% achieved 2 km cycling/day and in control group 8.9% (OR =7.8 [95%CI 4–15], *p* < 0.001). **+** The intervention group was more likely to comply with at least one of the treatment goals (either cycling 2 km/day or walking 10,000 steps/day) than the control group: 60.8% vs. 41.8% (OR = 2.2 [95% CI: 1.3–3.8, *p* = 0.003]). **+** Using bicycle to commute at least once/week during months 2–18 was 29.4% in intervention group and 8.0% in control group (*p* < 0.001). **0** Both intervention and control groups increased steps during follow-up (*p* < 0.001). Difference between groups (*p* = 0.10). **0** Of intervention group 47.5% reached 10,000 steps/day and of control group 39.3% (OR 1.2 [0.7–2.0], *p* = 0.50). **0** In intervention group 40.4% and in control group 47.4% (*p* > 0.05) commuted by walking during months 2–18. **0** During months 2–18, commuting by car was 25.2% in the intervention group and 28.9% in the control group (*p* > 0.05). **0** Commuting by public transport was 55.5% in both groups during months 2–18 (*p* > 0.05). **+** In whole group (intervention + control) a 34.1% decrease in number of commuting journeys by car per participant (*p* < 0.01). **+** In whole group (intervention + control) a 37.1% reduction in number of commuting journeys by public transport (*p* < 0.001).	Education, persuasion, enablement.

PA: physical activity; BMI: body mass index; BCW: Behaviour Change Wheel.

PA-related outcomes varied in the studies, between measures of volume (such as steps using pedometers, minutes/hours/days of exercise), intensity (mild, moderate, vigorous), measures of fitness (e.g. VO₂max, handgrip strength), total sedentary time or context-specific sedentary time (e.g. leisure time and school day sedentary time in children, and worktime, leisure time and weekend sedentary time in adults). Secondary outcomes included measurements in anthropometrics, subjective questionnaires and biomarkers such as blood lipids and blood pressure.

### Collating, summarising and reporting the results

The authors divided the intervention effects on PA, sedentary time or fitness into three categories: positive intervention effect, null results (no effect) and negative intervention effect. The decision on the category (effect) was based on the results at the final follow-up point, which varied across the studies. The interventions were considered to have positive effects if they included a statistically significant improvement (*p* ⩽ 0.05) in PA or fitness, or in the sedentary time outcome relative to the control arm. The negative intervention effects included a statistically significant decrease in PA or fitness, or an increase in sedentary time relative to the control arm. Finally, no effect signified a nonsignificant difference in change for PA, fitness or sedentary time between the groups. The interventions are presented in the Tables II and III and in detail in Supplemental Materials. Based on the intervention actions described in each study, Michie et al.’s [33] intervention functions were chosen by the authors of the current review.

The content of the interventions was summarized using the BCW approach [33]. The reported actions (content) in the interventions were allocated to one of the nine intervention function categories including education, persuasion, incentivization, coercion, training, restriction, environmental restructuring, modelling and enablement. Furthermore, the theoretical background of the interventions was listed if it was mentioned.

### Consultation

As Levac et al. [34] suggest consultation as the final part of scoping document methodology, senior researchers were consulted on the findings and methodological clearness.

## Results

Twelve studies representing 10 different interventions were accepted in the analysis according to the inclusion criteria. Six studies were school-based and focused on children aged 6 to 15, one study was family-based focusing on kindergarten-aged children and their parents, and five studies were conducted among working-age adult populations targeting either working life, leisure time or commuting. Eight of the studies were conducted in Finland, three in Denmark and one in Sweden.

### Children and adolescents

#### Family-based interventions

Only one family-based intervention met our inclusion criteria. The results of the intervention were presented in two different studies, one focusing on children [37], and the other focusing on adults [38]. The intervention was conducted as a randomized control trial (RCT) and the duration of the intervention and follow-up was 12 months.

For children, the PA outcome measures included gross motor tests (Körperkoordinationstest für Kinder and throwing and catching a ball), amount of moderate-to-vigorous PA (MVPA) and sedentary time measured by accelerometer. For adults, main outcomes included accelerometer-based PA and sedentary time.

In children, MVPA showed a small decrease (−0.08% [−0.24, 0.08]) in the intervention group, and a small increase in the controls (+0.08% [−0.08, 0.24]) with a significant group and time interaction (*p* < 0.05). The motor performance tests did not show any significant differences in changes between the groups. In adults, no significant differences were observed in changes in PA or sedentary time between the intervention and the control groups from baseline to 12-month follow-up.

The intervention was based on social cognitive theory and the theory of planned behaviour. The intervention content included education, persuasion and modelling according to the BCW functions [33].

#### School-based interventions

##### Format and duration

All school-based interventions were conducted as RCTs. The study times varied between one academic year [39,40], two academic years [41–43], and three academic years [44].

##### PA outcomes

MVPA was the most reported outcome measure to assess changes in PA, sedentary time was assessed in one study, leisure time PA and PA motivation were measured in one study, fitness tests (shuttle run, hand gip, Andersen test or vertical jump) were used as outcome measures in two studies and gross motor tests (walking backwards, hopping for height, jumping sideways, moving sideways, precision throw) were examined in one study. Both device-based and self-reported measurements of PA outcomes were used.

Studies reporting MVPA yielded positive intervention effects between groups in two studies [39,43], one intervention presented null effects [40] and one reported higher MVPA after follow-up in the control group [42]. For hand grip strength there was one study with a significant positive effect and one with a negative effect in the intervention compared with the control groups [41,44]. In relation to shuttle run, there was no effect (null result) in one study [44] whereas Christiansen et al. [41] demonstrated a positive effect. In the study by Rexen et al. [44] the intervention increased total development in the composite fitness *z*-score among 4th-grade children in the intervention group (difference 1.06 units [95%CI 0.48–1.65]), and the intervention benefit was biggest for children who were performing at the lower levels of physical fitness and PA at baseline.

Overall, from the six school-based interventions three used accelerometers to assess PA and/or sedentary time. Two studies used questionnaire methods to assess PA. Device-based measurements (accelerometer, measured anthropometrics, physical- and gross motor tests) were related to positive intervention effects in three of the four studies [41,43,44] and only one study failed to show any intervention effects [42]. Interventions using subjective assessment as a measurement method were inconclusive in relation to effect [39,40].

##### Other outcomes

One study assessed waist circumference, concluding no difference between the intervention and control groups at follow-up (0.2 cm, *p* = 0.91) [41]. Two studies measured PA motivation using the Motivational Climate in Physical Education Scale [40,39]. In addition, single studies reported a rating in a school yard PA environment [42], task- and ego-support [40], and task- and ego-orientation [39]. Regarding the school yard index, most components (different activities, fun challenging environment, plenty of space, hangout places, greenery and unfixed equipment) but not the rating ‘schoolyard is good for ball games’ showed a significantly larger increase in the intervention group compared with the control group (*p* ⩽ 0.02) [42]. In the study by Kokkonen et al. [40], task support in physical exercise (PE) increased (*p* = 0.008) and ego-support in PE decreased in the intervention group (*p* < 0.001). In the study by Gråsten et al. [39], the school-initiated PA programme had a weak negative effect on ego-orientation (β = −0.07, *p* < 0.05) and no effect was detected in task-orientation (β = 0.04, *p* < 0.001).

##### Theoretical background and intervention functions

Behaviour change theories or theoretical backgrounds were presented in five interventions (six studies), however the theoretical backgrounds varied between the interventions [39–43]. The intervention in all studies included actions related to more than one intervention function category as defined by the BCW [33]. The interventions in Haapala et al. [43] and in both Christiansen et al. studies [41,42] included the most varied set of intervention functions. Intervention actions related to education were included in all school-based interventions [39–44]. Actions related to enablement were included in four out of five interventions [39,41–44], actions of environmental restructuring in three out of five [39,41–43], actions of persuasion in 2 out of five [43,44], training-related actions in one out of five [40], modelling-related actions in one out of five [40] and restriction-related actions in one out of five interventions [41,42].

#### Adults

##### Format and duration

Interventions focusing on adult populations were all conducted among working-age people, three of them focusing on intervening directly or indirectly in the behaviour of the people in working life [38,45–47] and one focusing on increasing PA in commuting in general [48]. All of the selected studies were conducted as RCTs and the duration of the intervention and follow-up time varied between one calendar year [38,47], 18 months [48] and two calendar years [45,46].

##### PA outcomes

For the PA outcomes, two out of four interventions used device-based measurement methods (accelerometer) [38,45,46], one study combined device-based (pedometer, trip meter) and self-reported assessments (questionnaire, activity diary) [48] and one study used only self-reported assessment (questionnaire, notebook/diary) [47]. The outcomes included MET-hours per week of leisure time PA (calculated based on the questionnaire), habit of walking, habit of walking stairs, time spent in leisure time walking, time spent in daily walking, daily sitting time at work, daily sitting time outside work (all these outcomes were measured by a questionnaire, which was modified from the self-administered long version of the International Physical Activity Questionnaire for a typical week), accelerometer-based total daily light PA minutes, accelerometer-based total daily MVPA minutes, accelerometer-based total daily sedentary time, frequency of bicycling, frequency of commuting by bicycle (outcomes of frequency measured with a trip meter, self-reported mode of transport and a diary), frequency of commuting by walking (self-reported mode of transport and a diary), and step count per day (a pedometer). Furthermore, in one intervention the outcomes also included CRF measured as VO₂max (a sub-maximal cycle ergometer test).

One study observed significantly higher increases in VO₂max and leisure time PA in the intervention group in comparison to the control group [45,46]. Another study reported a significantly higher percentage of commuting by bicycle and reaching either 2 km cycling or 10,000 steps a day and using a bicycle for commuting in the intervention group compared with the control [48]. Neither of the two studies that followed changes in sedentary time [38,47] observed any significant intervention effects compared with the control. In the study by Pesola et al. [38] the purpose was to test the effectiveness of behavioural counselling aiming to reduce and break up sedentary time and to increase light-intensity PA during work and leisure time. In light of the intervention aims, the findings and differences between intervention and control groups were modest. Walking was reported in two studies, however the outcome definitions were different: Aittasalo et al. [47] reported time spent walking overall and for leisure, as well as the habit of walking in general, for leisure and climbing stairs, whereas Hemmingsson et al. [48] used pedometers to assess daily step counts. In the study by Aittasalo et al. [47], the purpose was to evaluate the effects of the intervention in promoting walking. However, neither of the studies reported positive intervention effects compared with the control groups. Overall, in two of the four interventions [38,47], borderline but otherwise nonsignificant differences between the intervention and control groups were observed as a result of the interventions.

##### Other outcomes

Other reported outcomes included workability [45,47], stress symptoms (SS) and mental resource (MR) indexes [46] at individual level, and reach, effectiveness, adoption, maintenance, costs and implementation of the intervention at administrative level [47]. Two of the four interventions examined anthropometrics as secondary outcomes [38,48].

Workability showed no change in one study [47], whereas Kettunen et al. [45] detected differences in the Work Ability Index (WAI) between the groups as a result of the intervention: the intervention group increased their mean WAI by 3% (*p* < 0.0001). The change in WAI between the intervention and control groups was significantly different during the intervention (baseline vs. 12 months, *p* = 0.028) and after the follow-up (*p* = 0.001). A slightly positive correlation between the changes in WAI was detected (*r* = 0.19, *p* < 0.01). According to Kettunen et al. [46] SS decreased by 16% and MR increased by 8% in the intervention group, while no significant changes were detected in the controls. In the study reporting administrative changes, the willingness of employees to participate was 29% (i.e. reach was 29%) and adoption and implementation succeeded as intended. In addition, the reported direct costs of the intervention were approximately EUR43 per participant in the intervention group [47].

Hemmingsson et al. [48] observed reductions in waist circumference and sagittal abdominal diameter in both the intervention and control groups and the effects were maintained until the end of follow-up. However, no changes in body weight were observed during the study period [48]. Biomarkers and anthropometrics were measured in the intervention by Pesola et al. [38], indicating favourable intervention effects on leg lean mass and ApoB/ApoA-1 ratio after follow-up.

##### Theoretical background and intervention functions

In three out of four interventions, a theoretical background to the intervention was mentioned; in some there was more than one theory behind the intervention. The theoretical backgrounds included the health action process approach [47], the theory of planned behaviour and motivational interview [38], and the transtheoretical model [48]. BCW intervention actions were related to education and persuasion in all five (four interventions) studies [38,45–48]. One intervention also included training actions [45], and one action related to enablement [48] and one to restriction [38].

## Discussion

This scoping review on long-term community PA interventions in the Nordic countries focused on increasing PA or fitness of the population. Most of the included interventions were from Finland (*n* = 8); none identified were from Norway or Iceland. Detailed descriptions of where the interventions took place were not included in the articles, which hindered discussions about the success or failure of an intervention mirroring the health and social factors of the intervention contexts.

The majority of interventions targeted children and adolescents, with school-based interventions being the most common approach. Working-age populations were targeted in several of the interventions; none including elderly participants met the inclusion criteria. Overall, the findings indicated that most PA interventions in the Nordic communities are built on theoretical frameworks, and include several behaviour change functions. However, there is considerable variation in the assessment of PA outcomes, and the intervention effects on PA were modest.

A primary observation was that PA assessments varied substantially between the included interventions. The studies using device-based measures used different devices, placed on slightly different locations (waist and hip). This hindered the comparison and summarizing of the findings between the included studies and studies in general. In the studies where device-based assessments of PA were applied, the results were slightly more positive in terms of increasing PA than in the studies where self-report methods were used. The efficacy of using questionnaires to measure both PA levels and sedentary behaviour is debatable, as self-reported PA or sitting times may include bias due to daily and personal variations in reporting, potentially leading to vague overall conclusions. In addition, questionnaires measuring PA levels may not be sensitive enough to detect a change in level, volume or intensity of PA. When using device-based measurements, data processing methods require more consistency in order to improve comparability between studies. One reason for the results being more favourable for device-assessed changes in PA than self-report methods could be due to devices being more sensitive to detecting small changes than, for example, questionnaires. Previous reviews on PA intervention studies have also noted the variability in assessment and operationalization of the main outcome as a significant limitation for the summation of the results and assessment of intervention effectiveness [49,50]. Following Ding et al. [51], we also call for improved and more standardized measurement of PA across future studies.

### Family-based interventions

We identified only one intervention targeting young children, and this was a family-based intervention where parents were also targeted. The parents were given targeted counselling in order to act as role models for their children. The intervention did not seem to be effective in increasing overall PA or decreasing the amount of sedentary time of children or adults, but it was effective in improving the motor control of the children. It is widely acknowledged that PA habits are formed in the early years of life indicating that behaviour models in the family play an important role in the development of PA habits among young children [52,53]. Families with young children are not necessarily physically inactive per se, nevertheless a continuous need for interventions among this target group in the Nordic environment remains.

### School-based interventions

The studies reporting on school-based interventions that met our inclusion criteria were conducted in Finland and Denmark. The Danish SPACE study [41,42] comprised improvements in the physical and organizational environment and educational activities in the school setting, increasing the total PA (MVPA min/day) of the students. The other Danish intervention consisted of extra PE lessons, (270 min/week compared with usual 90 min/week) [44] and was effective in increasing the fitness of older but not younger children. An important finding was, however, that increasing PE lessons showed effectiveness in relation to the fitness of the children who were least active at baseline [44]. This is of particular interest since interventions conducted in communities are challenged by the fact that they do not reach the target group (i.e. the least physically active or fit) well enough [30,31]. In one school-based intervention [42], higher MVPA levels were reached in the control arm than the intervention group. These results indicate the complex unpredictability of human behaviour and the importance of PA interventions for both groups.

Two of the Finnish school-based interventions targeted environmental restructuring [39,43] and one PE teaching [40]. Introducing the creative physical education model in PE teaching was not particularly effective in increasing the overall MVPA of the students [40]. However, embracing a task involving climate and the physical environment [39] increased the self-reported frequency of MVPA in children. The Move intervention, with the goal of creating a more active and pleasant school day through PA and tailored interventions, was effective in increasing the school day MVPA in primary grade students [43]. The programme was also found to contribute by decreasing school day sedentary time among the intervention participants when compared with the controls [43].

Based on the reviewed studies, school-based interventions in the Nordic countries were somewhat more effective in increasing PA when they included environmental restructuring and enabled more variety in the PA of school days. Similar results were reported by Crutzen [54] and Temple and Robinson [55] who concluded that the most successful PA interventions among school-aged and preschool-aged children mainly focused on environmental restructuring in the school setting.

### Working-age adults

All four interventions targeting working-age adults that met our inclusion criteria had a strong focus on individually tailored actions, despite being considered community interventions. All the interventions included actions related to education and persuasion, taking the form of lectures, distribution of information, one-to-one counselling, group counselling or personal goal-setting. Nevertheless, the effectiveness of the interventions was generally slight, with only two studies finding an improvement in PA or fitness in the intervention group compared with the controls. According to the review by Müller-Riemenschneider et al. [49], tailored exercise prescription strategies are the most promising approaches for long-term increases in PA and fitness in healthy adults. They also concluded that using combined intervention approaches such as goal-setting, problem-solving, self-monitoring, planning and incentives provide the strongest possibility of intervention effectiveness. This can also be seen in the current review, where studies by Kettunen et al. [45,46] and research by Hemmingsson et al. [48] included the most extensive actions and were the ones resulting in significant improvements in PA and fitness. However, both Kettunen et al. studies [45,46] provided training camps, and Hemmingsson et al. [48] provided participants with new bicycles, neither of which could be feasibly replicated in a larger population.

Previously, Torp and Vinje [56] reported workplace interventions in the Nordic countries to be individually focused rather than targeting the setting itself (i.e. workplace environment). The intervention by Aittasalo et al. [47] was delivered via occupational healthcare to workplaces, and targeted sedentary office workers. The intervention yielded small, albeit nonsignificant improvements in individual level PA and sedentary time, but the authors reported that at the 12-month follow-up, positive changes in the workplace environment favouring more physically active behaviours had been incorporated. This finding is noteworthy, because it emphasizes that we also need to change the customs and habits induced by society and the environment in order to facilitate a change at the individual level. Even if community, multicomponent interventions engaging different sectors of society and targeting different levels of influence are complex and time-consuming to undertake and report, such interventions are warranted for truly making changes in population-level PA [51].

All but one of the interventions conducted among the adult population were based on a theoretical background, and these interventions were applied to the everyday life of the participants [38,47,48]. Effective behavioural interventions should consider context, behavioural habits and mechanisms of change [57], and the transfer effect of the intervention to everyday life needs to be ensured. In the current review, the inclusion of a background theory or implementation to everyday life did not yield strong effects on PA behaviour change.

### Strengths and limitations of our study

The country inclusion criterion was narrowed to include the Nordic countries only, which have similar cultural, social and economic backgrounds [36] and would therefore make possible more comparable analyses on what intervention functions, methods or tools might be successful. This is not to say that the Nordic countries are homogenous and similar in health behaviours, as such [58] However, it is important to examine the interventions in an attempt to understand the underlying root causes of decreased PA and increased sedentary time to increase population health in these countries through potentially effective interventions that could be applicable throughout the Nordic countries. When it comes to PA interventions, all Nordic countries are facing similar challenges among their populations: increasingly unhealthy lifestyles, increasing noncommunicable disease rates and an aging population, hence the topic is very relevant globally [2–4].

A 12-month minimum of intervention duration was selected to ensure sufficient time to be able to gather information on long-term effectiveness of the interventions. We acknowledge that the duration of the studies included both the active intervention and follow-up periods and thus did not enable separate evaluations of behaviour maintenance. It is known that intervention effects on health behaviours are often strongest immediately after the active intervention and the achieved changes tend to diminish over time without additional support [57]. However, limiting the included studies to those with at least a 12-month duration is also a study strength, since the synthesizing of studies with similar durations better justifies the interpretation of the results than if the intervention periods varied in duration.

In addition, long intervention durations are also predisposed to high dropout rates. We considered dropout as one inclusion criterion, as high dropout during an intervention and/or follow-up affects the robustness of the evidence [59]. Allowing more variation in the dropout rate could have rendered more studies eligible for evaluation, which could have furthermore affected the conclusions of this review. By selecting a low- over high dropout rate, some bias might have been introduced due to the potential loss of inactive people. Including studies with higher dropout rates might have allowed us to investigate the characteristics of the people involved in those studies, namely the possibly high-risk populations. It was also challenging to deem the dropout for different studies since the reporting of participation rates varied considerably and not all studies included a flowchart or clear presentation of the participation numbers throughout their interventions.

The review is based on English-language peer-reviewed academic articles, leaving out a number of well-conducted interventions reported in native languages or in grey literature (such as national reports). When identifying the intervention functions according to the BCW and analysing the results, the authors of this review based the conclusions on their shared opinions, giving three different perspectives to the discussion; decisions were based on the frequent discussions among the team. It is acknowledged that other approaches to mapping interventions exist, however, the BCW framework was chosen as it is an easy-to-use, evidence-based, previously used [60] framework for organizing and collating different intervention actions.

For future interventions, assessment of PA outcomes and methodological choices needs to be considered carefully to increase comparability between studies. More uniform, standardized ways of PA data processing need to be focused on to achieve synthesis between interventions. In addition, more peer-reviewed studies reporting findings from long-term interventions are warranted. An important aspect for future studies will also be to examine the characteristics of the people dropping out of PA interventions, to increase understanding on this population.

## Conclusions

In conclusion, this scoping review summarized the characteristics and functions of long-term PA interventions conducted in communities in the Nordic countries. Based on our results, the intervention effects in communities were modest when attempting to influence PA behaviour. However, developing and applying community approaches in PA interventions is important, as even small increases in PA, fitness or changes to earlier unhealthy habits are crucial steps towards increasing PA at a population level among all age groups.

## Supplemental Material

sj-docx-1-sjp-10.1177_14034948211020599 – Supplemental material for The effects of long-term physical activity interventions in communities: Scoping review in the Nordic countries Click here for additional data file. Supplemental material, sj-docx-1-sjp-10.1177_14034948211020599 for The effects of long-term physical activity interventions in communities: Scoping review in the Nordic countries by Elsi H. Haverinen, Hanna M. Elonheimo, Hanna K. Tolonen, Pekka J. Jousilahti and Heini J.C. Wennman in Scandinavian Journal of Public Health

sj-docx-2-sjp-10.1177_14034948211020599 – Supplemental material for The effects of long-term physical activity interventions in communities: Scoping review in the Nordic countries Click here for additional data file. Supplemental material, sj-docx-2-sjp-10.1177_14034948211020599 for The effects of long-term physical activity interventions in communities: Scoping review in the Nordic countries by Elsi H. Haverinen, Hanna M. Elonheimo, Hanna K. Tolonen, Pekka J. Jousilahti and Heini J.C. Wennman in Scandinavian Journal of Public Health
