# Supplementary material for: The effects of long-term physical activity interventions in communities: Scoping review in the Nordic countries
Source: Scand J Public Health. 2021 Jun 28;50(2):272–86. doi: 10.1177/14034948211020599 (PMC8873971; doi:10.1177/14034948211020599)
Supplement: sj-docx-1-sjp-10.1177_14034948211020599 – Supplemental material for The effects of long-term physical activity interventions in communities: Scoping review in the Nordic countries [file sj-docx-1-sjp-10.1177_14034948211020599.docx]

| **Authors and year:** | **Country** | **Title and bibliographic details** | **Study subjects:** | **Description of the intervention:** | **Study duration (intervention and follow-up):** | **Results of physical activity (PA)** | **Other results** |
| --- | --- | --- | --- | --- | --- | --- | --- |
| Gråsten, Yli-Piipari, Watt, Jaakkola & Liukkonen, 2015 | Finland | Effectiveness of school-Initiated physical activity program on secondary school students’ physical activity participation  *J Sch Health* 2015; 85(2): 125-134. | N=847 (422 girls, 425 boys). Experimental condition school (n=208) and four control schools (n=639). Secondary school students aged 12-14 from two school districts. | The Sotkamo Physical Activity as Civil Skill Program was designed and launched to increase student`s physical activity by promoting supportive psychological and physical school environment. The intervention included task-involving climate and physical school environment treatments. The task-involving climate treatment consisted of supplementary teacher training and task-involving climate support in regular physical education classes in form of four workshops. The physical school environment treatment focused on recess activities including extended breaks, access to fitness hall and controlled ball games, as well as on developing the physical environment of the school and providing equipment. Two measurement phases were carried out using self-report questionnaires in April 2011 and 2012. | One academic year. | Intervention vs. control: 13.4% higher self-reported total MVPA level at follow-up. Effect sizes for intervention beta=0.19, p<0.001; R2=0.39. Self-reported MVPA of control students decreased. | Weak negative intervention effect on ego-orientation (beta=-0.07, p<0.05), and no influence on task-orientation (beta=0.04, p>0.05). R2task = .37, R2ego = .16. |
| Laukkanen, Pesola, Heikkinen, Sääkslahti & Finni, 2015 | Finland | Family-based cluster randomized controlled trial enhancing   physical activity and motor competence in 4-7 year-old children  *PLoS One* 2015; 10(10): e0141124. | N=103. Children aged 4-7 and their families, intervention group families (n=46) and control group families (n=45). | Parents in the intervention group families received tailored counseling based on the social cognitive theory and theory of planned behavior, with the goal to increase their children`s PA. Nine different behavior change techniques were used: providing instruction, providing information on consequences, prompting identification as a role model, providing general encouragement, providing information about others approval, prompting intention formation, progressive goal setting, prompting barrier identification, self-evaluation. The behavior change techniques were included in one or several parts of the counseling process: 1) a lecture, 2) individual face-to-face counseling and goal setting, and 3) counseling by phone.  Control group families did not receive any counseling. | One year. | Intervention group 0-12 months mean changes: sedentary time (%) 0.02 [-0.11; 0.15], light PA (%) 0.04 [-0.06; 0.15], moderate to vigorous PA (%) -0.08 [-0.24; 0.08], gross motor coordination (score) 35.28 [29.6; 41.0] (p<0.001). Control group 0-12 months mean changes: sedentary time (%) -0.10 [-0.22; 0.03], light PA (%) 0.06 [-0.04; 0.17], moderate to vigorous PA (%) 0.08 [-0.08; 0.24], gross motor coordination (score) 36.76 [30.97; 42.54]. (p<0.001).  Difference in change between groups (intervention vs. control): sedentary time (mean difference 0.11%, [-0.02 - +0.25], p= 0.106); light PA (mean difference –0.02%, [-0.13 - +0.09], p=0.285); moderate to vigorous PA (mean difference –0.16%, [-0.32% - +0.001%], p =0.033); gross motor coordination score (mean difference -1.47, [-9.52 - +6.58], p=0.737); throwing and catching a ball (p=0.984)  The intervention group had a steadier development of the KTK score when also the interaction with season was taken into account (p for group*time*season = 0.008). | BMI no significant change over time |
| Pesola, Laukkanen, Heikkinen, Sipilä, Sääslahti & Finni 2017 | Finland | Accelerometer-assessed sedentary work, leisure time and cardio-metabolic biomarkers during one year: effectiveness of a cluster randomized controlled trial in parents with a  sedentary occupation and young children  *PLoS One* 2017; 12(8): e0183299. | N=133. Office workers with young children, intervention group (n=71, age 36.6 years) and control group (n=62, 39.6 years). | The intervention group received a lecture within 2 weeks from baseline measurements, followed by two face-to-face discussions including contractually binding goal setting, and phone counseling (for one parent at a time) after 2 and 5 months. The intervention was based on previously identified effective behavior change techniques (providing instruction, providing information on consequences, prompting identification as a role model (to children), providing general encouragement, providing information about others approval, prompting intention formation, prompt specific goal setting, agree on behavioral contract, prompt review of behavioral goals, prompt barrier identification). Background theories included theory of planned behavior and motivational interview. After the baseline, there were measurements every 3 months until the one-year-follow-up.  The last six months of the intervention were similar to both the intervention and control groups: 9 and 12 months assessments without any counseling. After the completed follow-up assessments, the participants in the control group received a shortened version of the counseling. | One year. | The mean change in intervention group between baseline and 12months: total SED (min/16h) -1.0 [95% CI -20.4; +18.4]; total Light PA (min/16h) 3.2, [-15.5; +21.9]; total MVPA (min/16h) -1.7, [-6.6; +3.1]; total breaks per SED hour 0.2, [-0.4; +0.9]. Work time SED (min/8h) -6.7, [-22.1; +8.7], work time light PA (min/8h) 7.6, [-7; +22.3]; work time MVPA (min/8h) -0.8, [ -3.5; +1.9], work time breaks per SED hour 0.1, [-0.9; 1.1]. Leisure time SED (min/8h) 0.8, [-10.5; +12.1], leisure time light PA (min/8h) -1.2, [-12.1; +9.8], leisure time MVPA (min/8h) 0.9, [-3.1; +4.9], leisure time breaks per SED hour 0.3, [-0.6; +1.1]. Weekend SED (min/16h) -22.9, [-58.8; +13.0], weekend light PA (min/16h) 16.7, [-18.7; +51.5], weekend MVPA (min/16h) 7.4, [-4.7; +19.6], weekend breaks per SED hour 0.3, [-0.9, +1.5].  The mean change in control group between baseline and 12months: total SED (min/16h) 10.4, [-9.5; +30.2]; total Light PA (min/16h) -8.2, [-27.3, +10.9]; total MVPA (min/16h) -2.1, [-7.1, +2.8]; total breaks per SED hour 0.5, [-0.1, +1.1]. Work time SED (min/8h) -6.5, [-22.0; +8.9], work time light PA (min/8h) 7.5, [-7.1; +22.2]; work time MVPA (min/8h) -1.0, [-3.7, +1.6], work time breaks per SED hour 1.2, [+0.2; +2.2]. Leisure time SED (min/8h) 8.6, [-3.0; +20.2], leisure time light PA (min/8h) -4.9, [-16.1; 6.4], leisure time MVPA (min/8h) -3.6, [-7.7; +0.5], leisure time breaks per SED hour -0.3, [-1.2; +0.6]. Weekend SED (min/16h) 2.7, [-34.5; +39.8], weekend light PA (min/16h)-3.0, [-39.0; +33.0], weekend MVPA (min/16h) 0.1, [-12.2; +12.4], weekend breaks per SED hour 0.5, [-0.8; +1.8].  The difference in change between intervention and control groups until 12months: total SED (min/16hours) mean difference –11.4 [95% CI -39.0; +16.4], p=0.79) ; total light PA (min/16hours) 11.3, [-15.4; +38.1], p=0.84;  total MVPA (min/16hours) 0.4, [-6.5; +7.3], p=0.39;  total breaks per SED hour –0.3, [-1.2; +0.6], p=0.88;  worktime SED (min/8hours)–0.1, [-22.0; +21.7], p=0.95;  worktime light PA (min/8hours) 0.1, [-20.6; +20.8], p=0.98;  worktime MVPA (min/8hours) 0.2, [-3.6; +4.0], p=0.28;  breaks per SED hour –1.1, [-2.5; +0.4], p=0.70;  leisure time SED (min/8hours) –7.9, [-24.0; +8.3], p=0.26;  leisure time light PA (min/8hours) 3.7, [-12; +19.4], p=0.52;  leisure time MVPA (min/8hours) 4.5, [-1.2; +10.2], p=0.54;  leisure time breaks per SED hour 0.6, [-0.6; +1.8], p=0.53;  weekend SED (min/16hours) –25.6, [-77.2; +26.1], p=0.31;  weekend light PA (min/16hours) 19.7, [-30.4; +69.8], p=0.59;  weekend MVPA (min/16hours) 7.4, [-9.9; +24.7], p=0.22;  weekend breaks per SED hour –0.2, [-2.0; +1.6], p=0.16. | Significant differences in the change between intervention and control group from baseline to 12 months were observed in the following outcomes: Weight -0.95 (-1.76 to -0.13) p = 0.33, with change in control group 0.84 (0.25 to 1.43)** and in intervention group -0.11 (-0.67 to 0.46). BMI -0.28 (-0.55 to -0.01)* p = 0.41, with change in control group 0.28 (0.08 to 0.47)** and in intervention group 0.00 (-0.19 to 0.19). Arm fat mass (%) -0.09 (-0.17 to -0.02)* p = 0.50, with change in control group 0.12 (0.07 to 0.18)*** and in intervention group 0.03 (-0.02 to 0.08). Leg lean mass (%) 0.48 (0.18 to 0.77)** p = 0.11, with change in control group -0.44 (-0.65 to -0.22)*** and in intervention group 0.04 (-0.16 to 0.24). Total lean mass (%) 1.06 (0.29 to 1.83)**, p = 0.07, with change in control group 0.56 (-1.13 to 0.00) and in intervention group 0.50 (-0.03 to 1.02). Tot chol -0.13 (-0.32 to 0.06), p = 0.042, with change in control group 0.04 (-0.09 to 0.18) and in intervention group -0.09 (-0.22 to 0.04). HDL 0.00 (-0.10 to 0.09), p = 0.022, with change in control group -0.12 (-0.19 to -0.06)*** and in intervention group -0.13 (-0.19 to -0.06)***. ApoA1 (g/L) 0.05 (0.00 to 0.11)**, p = 0.60, with change in control group 0.00 (-0.04 to 0.03) and in intervention group 0.05 (0.01 to 0.09)**. ApoB/ApoA1 -0.04 (-0.07 to -0.01)**, p = 0.028, with change in control group 0.02 (0.00 to 0.04)* and in intervention -0.02 (-0.04 to 0.00)*.  In other secondary outcomes the difference in change between groups was nonsignificant. |
| Haapala, Hirvensalo, Kulmala, Hakonen, Kankaanpää, Laine, Laakso & Tammelin, 2017 | Finland | Changes in physical activity and sedentary time in the Finnish schools on the Move program: a quasi-experimental study  *Scand J Med Sci Sports* 2017; 27(11): 1442-1453. | N=319. Girls and boys, aged 7-15, grades 1-9. Four program schools and two reference schools. Intervention group (n=223) and control group (n=96). | The Finnish Schools on the Move program is a national action program to create a more active and pleasant school day through PA in Finnish comprehensive schools. Specific actions were not required from the schools, but the program featured support to the schools by disseminating best practices and ideas in national seminars and program webpages and providing opportunities for support from experienced local mentors. There was a bottom-up approach which enabled the schools to plan and implement their own individual plans to make the school day more physically active. | Two academic years. | The difference between intervention and control primary-grade schools at follow-up: School day moderate-to-vigorous PA (MVPA) (min/h) 1.14 [SE 0.28], p<0.001; School day sedentary time (SED) (min/h) -2.07, [SE 0.54], p<0.001; Leisure time MVPA (min/day) -5.77, [SE 2.78], p=0.038; Leisure time SED (min/h) 0.10, [SE 0.57], p=0.869; total MVPA (min/day) 1.40, [SE 3.57], p=0.695; total SED (min/h) -2.50, [0.69], p<0.001.  The difference in the change between intervention and control primary-grade schools: School day MVPA (min/h) (b =0.27, [SE 0.10], p=0.010, school day SED (min/h) (b -0.52, [SE 0.20], p=0.008), leisure time MVPA (min/day) (b=0.13, [0.99], p=0.894), leisure time SED (min/h) (b= 0.04, [SE 0.6], p=0.879), total daily MVPA (min/day) (b=1.33, [SE 0.26], 0.250), total SED (min/h)(b= -0.19, [0.35], p=0.584).  The difference between intervention and control lower secondary-grade schools at follow-up: School day MVPA (min/h) 0.13, [SE 0.19], p=0.481; School day SED (min/h) 1.52, [SE 0.55], p=0.006; Leisure time MVPA (min/day) -3.89, [SE 2.62], p=0.137; Leisure time SED (min/h) 0.39, [SE 0.60], p=0.518; total MVPA (min/day) -2.78, [SE 3.18], p=0.382; total SED (min/h) 0.53, [SE 0.49], p=0.285.  The difference in change between lower secondary-grade intervention and control schools: school day MVPA (min/h) (b=0.06, [SE 0.11], p= 0.561), school day SED (min/h) (b=-0.48, [0.26], p= 0.063), leisure time MVPA (min/day) (b=-1.31, [1.48], p=0.376), leisure time SED (min/h) (b= 0.10, [SE 0.27], p=0.709), total daily MVPA (min/day) (b= -0.65, [SE 1.68], p=0.698), total SED (min/h) (b= 0.33, [SE 0.224], p=0.162). | Not reported |
| Kettunen, Vuorimaa & Vasankari, 2014 | Finland | 12-mo intervention of physical exercise improved work ability - especially in subjects with low baseline work ability  *Int J Environ Res Public Health* 2014; 11(4): 3859-3869. | N=371. 338 in exercise group (212 women and 126 men) and 33 in control group (17 women and 16 men). Aged 20-60 (mean age women 44 and men 42 years). | The exercise group participated in a 12-month exercise program followed by 12-month follow-up without any coaching. The exercise program consisted of two-day training camps at the Sport Institute of Finland, at baseline, 4-, 8-, 12- and 24-months. Training camps constituted of measurements and supervision to exercise and exercise in groups. Between the training camps the exercise group received one to two supervised exercise sessions in a group per month and three to five unsupervised exercise sessions per week. Every participant had an individualized exercise program based on their estimated oxygen uptake (VO₂max). Exercise was monitored with Polar heart rate monitors and recorded with personal web-based exercise diaries that were followed by the group coach and used to adjust training if necessary.  The control group received no supervised exercise or program. | Two years (12-month supervised exercise program and 12-month follow-up without exercise couching). | VO2max (ml/kg/min) in intervention group at baseline= 32.1 +-0.7, after 12 months= 34.4 +-7.6 (+7%, p<0.0001), and after 24 months=33.6 +-7.7 (+5%, p<= 0.01). VO2max (ml/kg/min) in control group at baseline= 35.4 +-11.3, after 12 months= 34.4 +-10.9, after 24 months= 34.6 +-11.2. Not sign. different from baseline. Change in VOmax between groups significant at 12-months (p=0.008) and 24 months (p=0.015).  LTPA in intervention group 8.3 +- 8.8 and 14.2 +-47.2 MET-hours/week at baseline and 12 months. The self-reported LTPA of the intervention group increased by 71% (p=0.016) during the intervention. LTPA (MET-hours/week) in control group 7.7 +-10.1 at baseline and 7.9 +-10.2 at follow-up. | Work ability index in intervention group 41.4 +-4,7 and 42.8 (+-4.6) and 42.0 +-4.9 at baseline, 12 months and 24 months. Improvement 3% (p<0.001) at 12 months. In control group 42.5 +-4.9 and 41.7 +-4.8 and 40.7 +-5.3 at baseline, 12 months and 24 months. Decrease by 2% (p=0.066) at 12 months and 4% (p=0.003) at 24 months. |
| Kettunen, Vuorimaa & Vasankari, 2015 | Finland | A 12-month exercise intervention decreased stress symptoms and increased mental resources among working adults -results perceived after a 12-month follow-up  *Int J Occup Med Environ Health* 2015; 28(1): 157-168. | N=371. 338 in exercise group (212 women and 126 men) and 33 in control group (17 women and 16 men). Aged 20-60 (mean age women 44 and men 42 years). | The exercise group participated in a 12-month exercise program followed by 12-month follow-up. The exercise program consisted of two-day training camps at the Sport Institute of Finland, at baseline, 4-, 8-, 12- and 24-months. Between the training camps the exercise group received one to two supervised exercise sessions in a group per month and three to five unsupervised exercise sessions per week. Every participant had an individualized exercise program based on their estimated oxygen uptake (VO₂max). Exercise was monitored with Polar heart rate monitors and recorded with personal web-based exercise diaries that were followed by the group coach and used to adjust training if necessary.  The control group received no supervised exercise or program. Both the intervention and control group participated in all the measurements at the same time points (baseline, 4-, 8-, 12- and 24-month). | Two years (12-month supervised exercise program and 12-month follow-up without exercise couching). | VO2max (ml/kg/min) in intervention group at baseline= 32.1 +-0.7, after 12 months= 34.4 +-7.6 (+7%, p<0.0001), and after 24 months=33.6 +-7.7 (+5%, p<= 0.01). VO2max (ml/kg/min) in control group at baseline= 35.4 +-11.3, after 12 months= 34.4 +-10.9, after 24 months= 34.6 +-11.2. Not sign. different from baseline. Change in VOmax between groups significant at 12-months (p=0.008) and 24 months (p=0.015).  Intervention group LTPA from 8.3 +-8.8 MET/h/week at baseline to 13.7 +-45.2 MET/h/week at 12 months. +71% (p=0.016) from baseline to 12-months. In control group LTPA at baseline 7.7 +-10.1 MET/H/week and at 12 months 7.9 +10.9MET/h/week. | Stress symptom index in intervention group 14.4 +-3.3 at baseline and 12.1 +-3.1 at 12months and 12.6 +-3.0 at 24months. The decrease was -16% (p<0.0001) from baseline to 12 months and 13% (p<0.0001) from baseline to 24 months.  In control group the SSI 12.3 +- 3.8 at baseline and 12.1 +-3.0 at 12months and 12.9 +- 3.1 at 24 months. No sig. change was observed between baseline and follow-up timepoints. Mental resource in intervention group 6.3 +-1.8 at baseline and 5.8 +-1.6 at 12months and 5.9 +-1.9 at 24months. The change was +8% (p<0.001) from baseline to 12 months and 5% from baseline to 24 months (p=0.031). In control group mental resource was 5.7 +- 1.4 at baseline and 6.5 +-1.5 at 12months and 6.5 +- 2.3 at 24 months. No sig. change was observed between baseline and follow-up timepoints. |
| Kokkonen, Yli-Piipari, Kokkonen & Quay, 2019 | Finland | Effectiveness of a creative physical education intervention on elementary school students’ leisure-time physical activity  motivation and overall physical activity in Finland  *Eur Phy Educ Rev* 2019; 25(3): 796-815. | N=382. Fourth to six grade students (mean age 10.87) from two elementary schools were allocated to the creative physical education (CPE) intervention (n=196, mean age 10.84) and control “PE as usual” (n=186, mean age 10.90) groups. | Both of the schools followed the National Core Curriculum for Basic Education. Intervention school PE classes were based on the principles of the CPE model that promotes the everyday experiences of young people, their typical ways of being by embracing an ontological conception of child-centeredness.  Prior to the intervention, a two day seminar was held to inform and demonstrate the CPE model to the entire intervention school PE faculty. Six teachers volunteered to participate and two were selected as teacher champions of the intervention. Teacher champions organized supplemental workshops for the remaining school teachers. A CPE student workbook (in Finnish) and educational materials were provided to all teachers.  In the control school PE lessons consisted mainly of the movement skill aims of two specific traditional sports: Finnish baseball and soccer. | One year. | Overall PA (days/week) in intervention group 4.44 +- 1.38 and 4.13 +- 1.68 at baseline and follow up. Overall PA (days/week) in control group 5.54 +- 1.38 and 5.51 +- 1.29 at baseline and follow up. No sig. intervention effect on overall PA (F[2.306]=1.55, p=0.213, n2 <= 0.01) | Task-support (points) in intervention group 4.01 +-0.79 and 4.16 +-0.70 at baseline and follow up. Ego support (points) 2.77 +- 0.97 and 2.70 +- 1.02 at baseline and follow up. Task support (points) in control group 4.34 +- 0.57 and 4.01 +- 0.76 at baseline and follow up. Ego support (points) 2.76 +-0.96 and 3.13 +-1.00 at baseline and follow up.  Being in intervention group increased task support in PE (F[2,306]= 7.01, p=0.008, n2 =0.02) and decreased ego-support in PE (F[2,306] =14.95, p<0.001, n2=0.06). Task support after intervention 4.19 +- 0.05; 4.24 +- 0.05; 3.99 +- 0.05 in 4th, 5th and 6th grades. (p<0.05 between 4th and 5th compared to 6th). Ego support after intervention 2.67+-0.08; 2.73 +-0.07; 3.12 +-0.07 (p<0.05 for 4th and 5th compared to 6th).  Intervention effect together with pretest values explained 16% of students’ perceptions of task-supportive climate in PE, 25% of ego-supportive climate in PE and 8% of leisure time PA motivation. Students leisure time motivation had positive effect on overall PA (beta =0.17), explaining together with pretest overall PA 20% of overall PA at followup. Students task support perceptions had a positive effect on their leisure time PA motivation (b=0.15, R2=0.08). Neither task nor ego supportive climate in PE had effect on overall PA (beta task= 0.05; beta ego =-0.06). |
| Christiansen, Toftager, Boyle, Kristensen & Troelsen, 2013 | Denmark | Effect of a school environment intervention on adolescent adiposity and physical fitness.  *Scand J Med Sci Sports* 2013; 23(6): 381-389. | N=1348. 7 intervention schools (n=623) and 7 control schools (n=725). Aged 11-14 (mean age 12.6 years), grades 5-6. | Intervention targeting the physical and organizational school environment for noncurricular physical activity (SPACE) on adiposity, aerobic fitness, and musculoskeletal strength. The intervention comprised of improvements in the physical and organizational environment and mainly focused on PA during recess in combination with some educational activities. The multicomponent intervention was developed according to the social ecological framework. The intervention package consisted of 11 components that included a combination of physical environment changes and supportive organizational changes. The intervention schools were supported to implement as many components as possible, but full implementation was not required. PE classes were not subject to intervention, and they were continued with the usual practice of 1.5 to 2 h per week at all schools. | Two years. | Intervention group shuttle (m) run result 1005 (95%CI 985; 1026) and 1039 (1021; 1058) at baseline and follow up. Control group shuttle run (m) 1005 (990; 1020) and 1036 (1013; 1060) at baseline and follow up. Difference between intervention and control group in shuttle run at follow up 6m, (95%CI -20; 31m), p=0.43.  Intervention group handgrip (kg) 24.0 (23.4; 24.6) and 31.6 (30.7; 32.5) kg at baseline and follow up. Control group handgrip (kg) 23.2 (22.7; 23.7) and 32.1 (31.1 33.1) kg at baseline and follow up. Difference between groups in handgrip (kg) at follow up -1.1 (-2.2; 0.0), p=0.05.  No difference in intervention effect by overweight, low SES or non-sports club PA participation. Boys had 0.8kg smaller increase in handgrip in intervention than control group (boys/intervention: 34.9 kg, (95% CI 34.0; 35.8), and boys/comparison: 35.7 kg CI (95% 34.8; 36.5), p sex*intervention=0.036. | Waist circumference in intervention group 69.4 cm (68.5; 70.4) and 75.9 (75.0; 76.7) at baseline and follow up. Waist circumference in control group 69.1 (68.0; 71.3) and 76.1 (73.7; 78.5) at baseline and follow up. Difference between group at follow up 0.2 (-2.5; 2.8), p=0.91. Boys in the intervention group had a borderline significantly smaller increase in waist circumference on 0.6 cm compared to the boys in the comparison group (boys/intervention: 75.8 cm, CI 95% 74.0; 77.8 and boys/comparison: 76.4 cm CI 95% 74.5; 78.3) |
| Christiansen, Toftager, Pawlowski, Andersen, Ersbøll & Troelsen, 2017 | Denmark | Schoolyard upgrade in a randomized controlled study design--how are school interventions associated with adolescents' perception of opportunities and recess physical activity  *Health Educ Res* 2017; 32(1): 58-68. | N=1348. 7 intervention schools (n=623) and 7 control schools (n=725). Aged 11-14 (mean age 12.6 years), grades 5-6. | SPACE for physical activity study, a multicomponent school-based intervention study aimed at improving PA levels among adolescents. The intervention components consisted of schoolyard renovation, mandatory outdoor recess and increased adult supervision and support; 11 components aiming at the physical and organizational environment in three main areas: after school fitness program, active school transport and recess PA. All schools implemented components targeting recess PA.  Environmental changes targeting recess PA included general upgrade of existing outdoor areas for PA and a specially designed playground designed by architects and student represent. The organizational environment changes included education of teachers as recess kick-starters, mandatory outdoor recess and/or access to sports hall. Implementation of the intervention was finished at least 6 months before follow-up measurements. | Two years. | Total PA (MVPA min /day) in intervention school pupils at follow up 44.8 (SD 23.5) and in control school pupils 49.9 (SD 27.6), p=0.003 between groups. Recess PA (mean cpm) in intervention school pupils at follow up 714 (SD 427) and control school pupils 642 (SD 417), p=0.01 between groups, also observed at baseline. Recess time in intervention school pupils at follow up 259.5 (SD 113.9) and control school pupils 229.4 (SD 77.9) p<0.001 between groups.    There was a strong effect of sex(boys) on recess PA (335mean CPM, p<0.001) and negative effect of age (-47 mean CPM, p=0.012). One unit increase in schoolyard index related to 12% (81 mean cpm) increase in recess PA (all schools). | Rating (Likert scale) of different school yard activities at follow-up in intervention schools 3.85 (SD 0.32) and control schools 3.52 (SD 0.19), p<0.001 between groups.  Rating of fun and challenging in intervention schools 3.24 (SD 0.32) and control schools 3.07 (SD 0.1), p<0.001 between groups.  Rating of goodness for ballgames in intervention schools 3.97 (SD 0.28) and control schools 3.98 (SD 0.20) p=0.53 between groups.  Rating of space in intervention school 3.97 (SD 0.26) and control schools 4.00 (SD 0.19) p=0.02 between groups.  Rating of hang-out places in intervention schools 3.93 (SD 0.16) and control schools 3.86 (SD 0.18) p<0.001 between groups.  Rating of greenery in intervention schools 3.90 (SD 0.23) and control schools 3.99 (0.36) p<0.001 between schools.  Rating of unfixed equipment in intervention schools 3.45 (SD 0.53) and control schools 2.83 (SD 0.32) p<0.001 between groups.  In intervention schools rating of different activities and access to unfixed equipment increased significantly and in control schools all schoolyard variables decreased at follow-up compared with baseline. |
| Rexen, Ersbøll, Møller, Klakk, Wedderkopp & Andersen, 2015 | Denmark | Effects of extra school-based physical education on overall physical fitness development - the CHAMPS study DK  *Scand J Med Sci Sports* 2015; 25(5): 706-715. | N=1247. 10 public schools, preschool to fourth grade pupils. Six schools engaged in extra PE and four schools in normal PE, normal PE (n=536) and extra PE (n=711). | In the CHAMPS study DK, all primary schools in the municipality of Svendborg were called to participate in the project as schools with extra PE (270 min/week divided over at least three sessions per week). In order to safeguard optimal quality of the PE lessons, PE teachers and pedagogues participated in a 40-lesson course in “Age-related concepts of training”. Also a handbook was provided, which contained inspiration for the content of PE lessons, practical suggestions, and exercises. The concept comprised focus on gradual development of all-round fundamental motor skills for the pre-pubertal children through organized playing activities involving training of movement skills. | Three academic years. | Fitness composite z-score development across 2.5 years. In Cohort 0 normal PE group = 7.16 (95% CI 6.69-7.64), extra PE= 6.83 (95% CI 6.43-7.23), difference between groups -0.33 (95% CI -0.95; 0.29), p=0.29.  In cohort 1 normal PE group= 5.84 (5.40-6.29), in extra PE group=6.30 (5.93-6.68), difference between groups 0.46 (-0.12; 1.04) p=0.12.  In cohort 2, normal PE group= 4.74 (4.31-5.16), in extra PE group= 4.84 (4.46-5.21), difference between groups 0.10 (-0.47; 0.66), p=0.73.  In cohort 3, in normal PE group=4.59 (4.16-5.02), in extra PE school 5.11 (4.72-5.49), difference 0.52 (-0.06; 1.09), p=0.078.  In cohort 4, in normal PE school 4.62 (4.21-5.04), in extra PE school 5.69 (5.28-6.09), difference 1.06 (0.48; 1.65), p<0.001.  Total difference over 2,5 yrs in fitness z-score 0.47 (0.08-0.85), p=0.018 between normal PE and extra PE schools among children in lowest 50% of baseline fitness, favoring extra PE schools. |  |
| Hemmingsson, Uddén, Neovius, Ekelund & Rössner, 2009 | Sweden | Increased physical activity in abdominally obese women through support for changed commuting habits: a randomized clinical trial  *Int J Obes* 2009; 33: 645-652. | N=120. Intervention group (n=60), control group (n=60). Abdominally obese women aged 30-60; mean age in the intervention group 47.8 and in the control group 48.6. | Behavior change principles of both groups were based on the Trans-theoretical model. Focus was on three processes of change: raising awareness (increased understanding of the need for intervention), countering (replacing the unhealthy behavior with healthy behavior) and helping relationships (support from family and friends etc.).  The control group received standard care that included a low-intensity, pedometer-driven walking intervention with two 2-h group counselling sessions (baseline and 6 months). During the group sessions walking recommendations were given and daily routines for physical activity and an individual physical activity plan were promoted. After baseline participants were encouraged to gradually increase daily walking up to 5000 steps/day, other forms of exercise and utilizing the pedometer were encouraged. The goal was to reach 10 000 steps/day at end of follow-up. Participants already walking 10 000 steps/day were encouraged to maintain their steps or to increase by 5000 steps/day.  The intervention group received the standard care (described above) combined with a more intensive behavioral counselling package: 1) three individual 30 min sessions with a physician at baseline, 6 and 12 months including detailed physical activity prescriptions, 2) two added 2 hour group counselling sessions during the cycling season at 2 and 14 months and 3) a new bicycle with free of charge bicycle service. | 18 months. | Intervention group step count baseline= 8692 steps, 18 months =10 129 steps (change p<0.001). Control group baseline=8249 steps, 18months= 9086 steps (change p<0.001). Difference between groups p=0.10. In intervention group 38.7 % achieved 2 km cycling/day and in control group 8.9 % (odds ratio =7.8 ((95% CI 4-15, p< 0.001)).  Of intervention group 47.5 % reached 10 000 steps/day and of control group 39.3%, OR 1.2 (0.7-2.0) p=0.50.  The intervention group was more likely to comply with at least one of the treatment goals (either cycling ⩾2 km/d or walking 10 000 steps per day) than the control group: 60.8 vs 41.8% (odds ratio=2.2 (95% CI: 1.3 to 3.8, P=0.003)).  Using bicycle to commute at least once/week during months 2-18 was 29.4% in intervention group and 8.0% in control group (p<0.001). In intervention group 40.4% and in control group 47.4% (p>0.05) commuted by walking during months 2-18. During months 2–18, commuting by car was 25.2% in the intervention group and 28.9% in the control group (P>0.05). Commuting by public transport were 55.5% in both groups during the same time period (P>0.05). In whole group a 34.1% decrease in number of commuting journeys by car per participant (P<0.01), and a 37.1% reduction in number of commuting journeys by public transport (P<0.001). | Waist circumference change in intervention group -2.1 cm (-3.4; -0.8) and in control group -2.6 cm (-4.0; -1.2) from baseline to 18 months. Body weight change in intervention group -0.4kg (-1.6; 0.7) and in control group -0.3 (-1.2; 0.7) between baseline and 18 months. Sagittal abdominal diameter change in intervention group -1.0cm (-1.3; -0.7) and in control group -1.1cm (-1.4; -0.8) from baseline to 18 months. |
| Aittasalo, Rinne, Pasanen, Kukkonen-Harjula & Vasankari, 2012 | Finland | Promoting walking among office  employees - evaluation of a randomized  controlled intervention with pedometers  and e-mail messages  *BMC Public Health* 2012; 12: 403. | N=241. Voluntary and insufficiently physically active office-employees were randomized to a pedometer (STEP, n=123) and a comparison group (COMP, n=118). Mean age in STEP-group was 44.1 and in COMP-group 45.3. 87 % of STEP-group participants were women whereas in COMP-group 78 % were women. | STEP comprised of one group meeting, log-monitored pedometer-use and six email messages from occupational health care units (OHC). COMP took part in data collection. The employees of STEP took part in a 6-month intervention comprising of: 1) a 1-hour preliminary meeting in each worksite held by a researcher and providing information on the intervention and on health benefits and recommendations of PA and walking. 2) Self-monitoring of PA with the pedometer and logbook. 3) Monthly e-mail message from OHC.  Personal walking goals were set by adding daily number of steps during five days of a week.  In COMP group only data was collected. After 12-month follow-up, however, a 1-hour meeting was offered to each worksite to provide feedback to all employees and to offer the COMP-participants pedometers, logbooks and walking leaflets. | One year. | Walking stairs in intervention group at baseline 38 min (SD 45) and follow up 63 min (SD 63). In control group at baseline 46 min (SD 100) and follow up 56 min (SD 79). Nonsignificant difference in change between groups in weekly minutes of walking stairs at 12 months (geometric mean ratio [GMR] 1.18 [0.91-1.53])  Percentage (%) walking stairs in intervention group at baseline 86% and follow up 93% and in control group 81% and 79% (intervention vs. control OR 2.24, 0.94 – 5.31).  Leisure time walking in intervention group at baseline 72 min (SD 96) and follow up 115min (SD 130). In control group at baseline 68 min (SD 125) and follow up 79min (SD 102). Nonsignificant difference in change between groups in weekly minutes of leisure time walking at 12 months (GMR 1.21, [0.98-1.59]). %walkers for leisure in intervention group at baseline 74% and follow up 87% and in control group at baseline 71% and follow up 76% (OR 2.07, 0.99 – 4.34).  Baseline walking at work in intervention group 144 min (SD 209) and control group 157 min (SD 236). Follow up in intervention group 172 min (SD 191) and control group 145 min (+-155). Nonsignificant difference in change between groups in weekly minutes of walking at work (GMR 1.13, [0.92-1.38]) %Walking at work in intervention group baseline 98% and follow up 99% and in control group baseline 96% and follow up 96% (int. vs. control OR 2.39, 0.15 – 37.3).  Walking for transportation in intervention group at baseline 115 min (SD 172) and follow up 170 min (SD 298). In control group at baseline 134 min (SD 168) and follow up 127min (SD 172). Nonsignificant difference in change between groups in weekly minutes of walking for transportation (GMR 1.03, [0.77-1.39]).  %walking for transportation in intervention group at baseline 77% and follow up 83% and in control group 81% and 79% (int. vs. cont OR 1.57, 0.68 – 3.61).  Total walking in intervention group at baseline 370 min (SD 311) and follow up 521 min (+-468). In control group at baseline 400 min (SD 401) and follow up 395 min (SD 319). Difference at 12months GMR= 1.25 (0.98; 1.59).  Sitting during working day in intervention group at baseline 544 min (SD 152) and follow up 477 min (SD 159). In control group at baseline 554 min (SD 161) and follow up 510 min (SD 183). Mean difference at 12 months -9min (-56; +37). Sitting during non-working day in intervention group at baseline 347 min (SD 165) and follow up 300min(SD 153) and control group at baseline 382 min(SD 180) and follow up 347min(SD 165). Mean difference at 12months -9 (-52; +33). | 100% of participating occupational health care units and worksites carried out the intervention.  Subjective work ability difference between intervention and control groups at 12 months 0.3 (-0.1; 0.6).  At the 12-month follow-up, 60% of the intervention group had used pedometers regularly and 37% irregularly during the 6-month intervention. The corresponding percentages regarding the logbooks were 46 and 47. Considering the e-mails, they reached 98% of the participants at 2 months and 99% at 6 months. According to the participants, the mean number of messages received was 2 (SD 0.7) at 2 months and 5 (1.1) at 6 months. At 6 months, 80% of the participants reported having read the messages. Regarding the setting level, based on the interviews of the employer-representatives, actions to promote PA during the 6 months after the cessation of the intervention had been taken at nine (45%) worksites. Indications of some traces of the intervention were seen in 6 (30%) worksites. The direct costs of the intervention were 5337 Euros, which contributed to approximately 43 Euros per participant in STEP. |
