# Supplementary material for: The effects of long-term physical activity interventions in communities: Scoping review in the Nordic countries
Source: Scand J Public Health. 2021 Jun 28;50(2):272–86. doi: 10.1177/14034948211020599 (PMC8873971; doi:10.1177/14034948211020599)
Supplement: sj-docx-2-sjp-10.1177_14034948211020599 – Supplemental material for The effects of long-term physical activity interventions in communities: Scoping review in the Nordic countries [file sj-docx-2-sjp-10.1177_14034948211020599.docx]

Additional search Medline, focus on Nordic countries. 29.8.2019

1 ("physical activity" or "physical fitness" or sports or "sporting activit*" or exercise or inactivity or "lack of exercise" or "low physical activity" or "lack of physical activity" or "minimal physical activity" or "insufficient physical activity" or "lack of exercise" or "little exercise" or "no exercise" or "physical* passiv*" or "inactive lifestyle" or sedentary or sitting or "stationary behav*" or "Stationary bout*" or "Stationary interruption*" or "Stationary break*" or "excessive stationary" or "stationary time" or "movement behav*" or "passive lifestyle" or "screen time").ti,ab. or Sedentary Behavior/ or physical activity/ or exp exercise/ or exp sports/ or youth sports/ or "physical fitness"/ or "Sports and recreational facilities"/ or "physical education and training"/ or Screen Time/ (528065)

2 (promot* or prevent*).ti. or ("health promot" or "disease prevention" or "public health prevent*" or "preventive medicine" or "preventive health" or "primary prevent*" or "primordial prevent*").ab,kf. or Preventive Medicine/ or Preventive Health Services/ or Health Promotion/ or Primary Prevention/ or Public Health/ (614934)

3 pc.fs. (1238744)

4 2 or 3 (1633233)

5 ("expert* by experience" or "health information" or "public information" or "information steering" or governance or "community policy" or "community program*" or "community intervention*" or "local policy" or "local policies" or "community program*" or "community intervention*" or "community-based intervention*" or "community-based program*" or "community health intervention*" or "community health program*" or "community health" or "community environment" or "multilevel intervention*" or "public health program*" or "health planning" or "health policy" or "built environment*" or "environmental design" or "environmental health" or "social environment" or "environment* planning" or "urban planning" or "city planning" or "rural planning" or "town planning" or "recreational facilities" or "sport* facilities" or "exercise facilities" or "school based" or "school health" or "school program*" or "school intervention*" or "school environment" or "school setting*" or curricul* or extracurric* or "day care" or kindergarten* or nursery or nurseries or "early childhood education" or "family center*" or "family centre*" or "youth centre*" or "youth center*" or outreach or "third sector" or volunteers or NGO* or "Non-governmental organi*" or church* or parish* or congregation or "public intervention*" or "public program" or "voluntary service*" or "community network*" or "voluntary organisation*" or "neighborhood association*" or "civic association*" or "voluntary organization*" or "social media" or "social marketing").ti,ab. or consumer health information/ or Social Participation/ or Community Participation/ or Social Medicine/ or Built Environment/ or Environment design/ or Healthy People Programs/ or Regional Health Planning/ or City Planning/ or "Environment and Public Health"/ or Health Plan Implementation/ or Policy making/ or Health Policy/ or Public policy/ or Government Programs/ or Population-Based Planning/ or Community-Based Planning/ or Community medicine/ or Community Networks/ or "sports and recreational facilities"/ or Recreation/ or National health programs/ or School health services/ or Curriculum/ or Voluntary Health Agencies/ or Social Media/ or Social Marketing/ or Child Care/ or Child Day Care Centers/ or Nurseries/ or Community Health Planning/ or "Legislation as Topic"/ or Government Regulation/ (657185)

6 (finnish or scandinavia* or "nordic countries" or "nordic region" or swed* or denmark* or danish or norw* or iceland* or fareo* or greenland*).ti,ab,sh,kf,ia. (252661)

7 ("systematic review" or "scoping review" or meta-analysis or "randomized controlled trials as topic" or RCT or "randomized trial" or "randomised trial" or "randomised controlled" or "randomized controlled" or "randomized clinical" or "randomised clinical" or cohort* or cct or "controlled trial" or "controlled clinical" or follow-up or baseline or longitudinal or "before and after" or "before-after" or "parallel treatment" or "parallel group").ti,ab,pt,sh. or cost-benefit analysis/ or Program Evaluation/ or Health Impact Assessment/ or cohort effect/ or Cohort studies/ or Implementation studies/ or "Controlled Before-After Studies"/ or "Follow-up Studies"/ or Clinical Trial/ or Treatment Outcomes/ or "Outcome and Process Assessment"/ or Controlled Before-After Studies/ or Comparative Studies/ or Evaluation Studies/ or Outcome Assessment/ or Longitudinal studies/ (4851158)

8 Social Change/ (16730)

9 7 or 8 (4866438)

10 1 and 4 and 5 and 6 and 9 (140)

First round search, Medline 4.6.2019

1 ("physical activity" or "physical fitness" or sports or "sporting activit*" or exercise or inactivity or "lack of exercise" or "low physical activity" or "lack of physical activity" or "minimal physical activity" or "insufficient physical activity" or "lack of exercise" or "little exercise" or "no exercise" or "physical* passiv*" or "inactive lifestyle" or sedentary or sitting or "stationary behav*" or "Stationary bout*" or "Stationary interruption*" or "Stationary break*" or "excessive stationary" or "stationary time" or "movement behav*" or "passive lifestyle").ti,ab. or Sedentary Behavior/ or physical activity/ or exp exercise/ or exp sports/ or youth sports/ or "physical fitness"/ or "Sports and recreational facilities"/ or "physical education and training"/ (519857)

2 (promot* or prevent*).ti. or ("health promot" or "disease prevention" or "public health prevent*" or "preventive medicine" or "preventive health" or "primary prevent*" or "primordial prevent*").ab,kf. or Preventive Medicine/ or Preventive Health Services/ or Health Promotion/ or Primary Prevention/ or Public Health/ (606630)

3 pc.fs. (1227923)

4 2 or 3 (1616231)

5 ("health education" or "health information" or "public information" or "information steering" or governance or "community policy" or "community program*" or "community intervention*" or "community-based intervention*" or "community-based program*" or "community health intervention*" or "community health program*" or "community health" or "community environment" or "multilevel intervention*" or "public health program*" or "health planning" or "health policy" or "built environment*" or "environmental design" or "environmental health" or "social environment" or "environment* planning" or "urban planning" or "city planning" or "rural planning" or "town planning" or "recreational facilities" or "sport* facilities" or "exercise facilities" or "school based" or "school health" or "school program*" or "school intervention*" or "school environment" or "school setting*" or curricul* or "day care" or kindergarten* or nursery or nurseries or "early childhood education" or "family center*" or "family centre*" or "youth centre*" or "youth center*" or outreach or "third sector" or NGO* or "Non-governmental organi*" or "public intervention*" or "public program" or "voluntary service*" or "community network*" or "voluntary organisation*" or "neighborhood association*" or "civic association*" or "voluntary organization*" or "social media" or "social marketing").ti,ab. or health education/ or consumer health information/ or patient education as topic/ or Social Medicine/ or Built Environment/ or Environment design/ or Healthy People Programs/ or Regional Health Planning/ or City Planning/ or Health Plan Implementation/ or Policy making/ or Health Policy/ or Public policy/ or Government Programs/ or Population-Based Planning/ or Community-Based Planning/ or Community medicine/ or Community Networks/ or "sports and recreational facilities"/ or Recreation/ or National health programs/ or School health services/ or Curriculum/ or Voluntary Health Agencies/ or Social Media/ or Social Marketing/ or Child Care/ or Child Day Care Centers/ or Nurseries/ (575478)

6 (Europ* or Finland* or finnish or scandinavia* or "nordic countries" or "nordic region" or swed* or denmark* or danish or norw* or iceland* or scotland* or scottish or uk or "united kingdom*" or Canad* or Holland* or dutch or Netherlands or "New zealand*" or Australia*).ti,ab,sh. (1362758)

7 ("systematic review" or "scoping review" or meta-analysis or "randomized controlled trials as topic" or RCT or "randomized trial" or "randomised trial" or "randomised controlled" or "randomized controlled" or "randomized clinical" or "randomised clinical" or cohort*).ti,ab,pt,sh. (1099186)

8 1 and 4 and 5 and 6 and 7 (524)

Additional search, focus on Nordic countries Cinahl, PsycInfo, Eric 30.8.2019

S6 S1 AND S2 AND S3 AND S4 AND S5 (77)

S5 ("systematic review" or "scoping review" or meta-analysis or "randomized controlled trials as topic" or RCT or "randomized trial" or "randomised trial" or "randomised controlled" or "randomized controlled" or "randomized clinical" or "randomised clinical" or cohort* or cct or "controlled trial" or "controlled clinical" or follow-up or baseline or longitudinal or "before and after" or "before-after" or "parallel treatment" or "parallel group" or outcome* assessment or "parallel group" or "parallel treatment" or "clinical trial" or "implementation study" or "intervention study" or "treatment outcome" or "policy evaluation" or "evaluation study" or "comparative study") (1,558,553)TI ( promot* or prevent* ) OR AB ( "health promot" or "disease prevention" or "public health prevent*" or "preventive medicine" or "preventive health" or "primary prevent*" or "primordial prevent*") OR SU ( ("health promot" or "disease prevention" or "public health prevent*" or "preventive medicine" or "preventive health" or "primary prevent*" or "primordial prevent*" or “public health”) (322,303)

S4 TI (Finland* or finnish or scandinavia* or "nordic countries" or "nordic region" or swed* or denmark* or danish or norw* or iceland* or Faeroe* or Greenland*) OR AB(Finland* or finnish or scandinavia* or "nordic countries" or "nordic region" or swed* or denmark* or danish or norw* or iceland* or Faeroe* or Greenland*) OR SU(Finland* or finnish or scandinavia* or "nordic countries" or "nordic region" or swed* or denmark* or danish or norw* or iceland* or Faeroe* or Greenland*) (161,778)

S3 ("expert* by experience" or "health information" or "public information" or "information steering" or governance or "community policy" or "community program*" or "community intervention*" or "local policy" or "local policies" or "community program*" or "community intervention*" or "community-based intervention*" or "community-based program*" or "community health intervention*" or "community health program*" or "community health" or "community environment" or "multilevel intervention*" or "public health program*" or "health planning" or "health policy" or "policy planning" or "capacity building" or "built environment*" or "environmental design" or "environmental health" or "social environment" or "environment* planning" or "urban planning" or "city planning" or "rural planning" or "town planning" or "recreational facilities" or "sport* facilities" or "exercise facilities" or "school based" or "school health" or "school program*" or "school intervention*" or "school environment" or "school setting*" or curricul* or extracurric* or "day care" or kindergarten* or nursery or nurseries or "early childhood education" or "family center*" or "family centre*" or "youth centre*" or "youth center*" or outreach or "third sector" or volunteers or NGO* or "Non-governmental organi*" or church* or parish* or congregation or "public intervention*" or "public program" or "voluntary service*" or "community network*" or "voluntary organisation*" or "neighborhood association*" or "civic association*" or "voluntary organization*" or "social media" or "social marketing") (1,246,658)

S2 TI ( promot* or prevent* ) OR AB ( "health promot" or "disease prevention" or "public health prevent*" or "preventive medicine" or "preventive health" or "primary prevent*" or "primordial prevent*") OR SU ( (prevent* or promot* or "health promot" or "disease prevention" or "public health prevent*" or "preventive medicine" or "preventive health" or "primary prevent*" or "primordial prevent*" or “public health”) (851,281)

S1 TI("physical activity" or "physical fitness" or sports or "sporting activit*" or exercise or inactivity or "lack of exercise" or "low physical activity" or "lack of physical activity" or "minimal physical activity" or "insufficient physical activity" or "lack of exercise" or "little exercise" or "no exercise" or "physical* passiv*" or "inactive lifestyle" or sedentary or sitting or "stationary behav*" or "Stationary bout*" or "Stationary interruption*" or "Stationary break*" or "excessive stationary" or "stationary time" or "movement behav*" or "passive lifestyle" or "screen time") OR SU("physical activity" or "physical fitness" or sports or "sporting activit*" or exercise or inactivity or "lack of exercise" or "low physical activity" or "lack of physical activity" or "minimal physical activity" or "insufficient physical activity" or "lack of exercise" or "little exercise" or "no exercise" or "physical* passiv*" or "inactive lifestyle" or sedentary or sitting or "stationary behav*" or "Stationary bout*" or "Stationary interruption*" or "Stationary break*" or "excessive stationary" or "stationary time" or "movement behav*" or "passive lifestyle" or "screen time") (292,402)

Cinahl, PsycInfo, Eric 5.6.2019 (First round search)

( TI ( ("physical activity" or "physical fitness" or sports or "sporting activit*" or exercise or inactivity or "lack of exercise" or "low physical activity" or "lack of physical activity" or "minimal physical activity" or "insufficient physical activity" or "lack of exercise" or "little exercise" or "no exercise" or "physical* passiv*" or "inactive lifestyle" or sedentary or sitting or "stationary behav*" or "Stationary bout*" or "Stationary interruption*" or "Stationary break*" or "excessive stationary" or "stationary time" or "movement behav*" or "passive lifestyle") ) OR AB ( ("physical activity" or "physical fitness" or sports or "sporting activit*" or exercise or inactivity or "lack of exercise" or "low physical activity" or "lack of physical activity" or "minimal physical activity" or "insufficient physical activity" or "lack of exercise" or "little exercise" or "no exercise" or "physical* passiv*" or "inactive lifestyle" or sedentary or sitting or "stationary behav*" or "Stationary bout*" or "Stationary interruption*" or "Stationary break*" or "excessive stationary" or "stationary time" or "movement behav*" or "passive lifestyle") ) OR SU ( ("physical activity" or "physical fitness" or sports or "sporting activit*" or exercise or inactivity or "lack of exercise" or "low physical activity" or "lack of physical activity" or "minimal physical activity" or "insufficient physical activity" or "lack of exercise" or "little exercise" or "no exercise" or "physical* passiv*" or "inactive lifestyle" or sedentary or sitting or "stationary behav*" or "Stationary bout*" or "Stationary interruption*" or "Stationary break*" or "excessive stationary" or "stationary time" or "movement behav*" or "passive lifestyle") ) ) AND ( TI ( promot* or prevent* ) OR AB ( ("health promot" or "disease prevention" or "public health prevent*" or "preventive medicine" or "preventive health" or "primary prevent*" or "primordial prevent*") ) OR SU ( ("health promot" or "disease prevention" or "public health prevent*" or "preventive medicine" or "preventive health" or "primary prevent*" or "primordial prevent*") ) ) AND ( TI ( ("health education" or "health information" or "public information" or "information steering" or governance or "community policy" or "community program*" or "community intervention*" or "community-based intervention*" or "community-based program*" or "community health intervention*" or "community health program*" or "community health" or "community environment" or "multilevel intervention*" or "public health program*" or "health planning" or "health policy" or "built environment*" or "environmental design" or "environmental health" or "social environment" or "environment* planning" or "urban planning" or "city planning" or "rural planning" or "town planning" or "recreational facilities" or "sport* facilities" or "exercise facilities" or "school based" or "school health" or "school program*" or "school intervention*" or "school environment" or "school setting*" or curricul* or "day care" or kindergarten* or nursery or nurseries or "early childhood education" or "family center*" or "family centre*" or "youth centre*" or "youth center*" or outreach or "third sector" or NGO* or "Non-governmental organi*" or "public intervention*" or "public program" or "voluntary service*" or "community network*" or "voluntary organi*" or "neighborhood association*" or "civic association*" or "social media" or "social marketing") ) OR AB ( ("health education" or "health information" or "public information" or "information steering" or governance or "community policy" or "community program*" or "community intervention*" or "community-based intervention*" or "community-based program*" or "community health intervention*" or "community health program*" or "community health" or "community environment" or "multilevel intervention*" or "public health program*" or "health planning" or "health policy" or "built environment*" or "environmental design" or "environmental health" or "social environment" or "environment* planning" or "urban planning" or "city planning" or "rural planning" or "town planning" or "recreational facilities" or "sport* facilities" or "exercise facilities" or "school based" or "school health" or "school program*" or "school intervention*" or "school environment" or "school setting*" or "curricul*or day care" or kindergarten* or nursery or nurseries or "early childhood education" or "family center*" or "family centre*" or "youth centre*" or "youth center*" or outreach or "third sector" or NGO* or "Non-governmental organi*" or "public intervention*" or "public program" or "voluntary service*" or "community network*" or "voluntary organisation*" or "neighborhood association*" or "civic association*" or "voluntary organization*" or "social media" or "social marketing") ) OR SU ( ("health education" or "health information" or "public information" or "information steering" or governance or "community policy" or "community program*" or "community intervention*" or "community-based intervention*" or "community-based program*" or "community health intervention*" or "community health program*" or "community health" or "community environment" or "multilevel intervention*" or "public health program*" or "health planning" or "health policy" or "built environment*" or "environmental design" or "environmental health" or "social environment" or "environment* planning" or "urban planning" or "city planning" or "rural planning" or "town planning" or "recreational facilities" or "sport* facilities" or "exercise facilities" or "school based" or "school health" or "school program*" or "school intervention*" or "school environment" or "school setting*" or curricul* or "day care" or kindergarten* or nursery or nurseries or "early childhood education" or "family center*" or "family centre*" or "youth centre*" or "youth center*" or outreach or "third sector" or NGO* or "Non-governmental organi*" or "public intervention*" or "public program" or "voluntary service*" or "community network*" or "voluntary organi*" or "neighborhood association*" or "civic association*" or "social media" or "social marketing") ) ) AND ( TI ( (Europ* or Finland* or finnish or scandinavia* or "nordic countries" or "nordic region" or swed* or denmark* or danish or norw* or iceland* or scotland* or scottish or uk or "united kingdom*" or Canad* or Holland* or dutch or Netherlands or "New zealand*" or Australia*) ) OR AB ( (Europ* or Finland* or finnish or scandinavia* or "nordic countries" or "nordic region" or swed* or denmark* or danish or norw* or iceland* or scotland* or scottish or uk or "united kingdom*" or Canad* or Holland* or dutch or Netherlands or "New zealand*" or Australia*) ) OR SU ( (Europ* or Finland* or finnish or scandinavia* or "nordic countries" or "nordic region" or swed* or denmark* or danish or norw* or iceland* or scotland* or scottish or uk or "united kingdom*" or Canad* or Holland* or dutch or Netherlands or "New zealand*" or Australia*) ) ) AND ( TI ( ("systematic review" or "scoping review" or meta-analysis or "randomized controlled trials as topic" or RCT or "randomized trial" or "randomised trial" or "randomised controlled" or "randomized controlled" or "randomized clinical" or "randomised clinical" or cohort*) ) OR AB ( ("systematic review" or "scoping review" or meta-analysis or "randomized controlled trials as topic" or RCT or "randomized trial" or "randomised trial" or "randomised controlled" or "randomized controlled" or "randomized clinical" or "randomised clinical" or cohort*) ) OR PT ( ("systematic review" or "scoping review" or meta-analysis or "randomized controlled trials as topic" or RCT or "randomized trial" or "randomised trial" or "randomised controlled" or "randomized controlled" or "randomized clinical" or "randomised clinical" or cohort*) ) OR SU ( ("systematic review" or "scoping review" or meta-analysis or "randomized controlled trials as topic" or RCT or "randomized trial" or "randomised trial" or "randomised controlled" or "randomized controlled" or "randomized clinical" or "randomised clinical" or cohort*) ) ) (135)

Cochrane Library of systematic reviews 5.6.2019

prevention or promotion in Keyword AND "physical activity" or "physical fitness" or sports or "sporting activit*" or exercise or inactivity or "lack of exercise" or "low physical activity" or "lack of physical activity" or "minimal physical activity" or "insufficient physical activity" or "lack of exercise" or "little exercise" or "no exercise" or "physical* passiv*" or "inactive lifestyle" or sedentary or sitting or "stationary behav*" or "Stationary bout*" or "Stationary interruption*" or "Stationary break*" or "excessive stationary" or "stationary time" or "movement behav*" or "passive lifestyle" (34)

Additional search, focus on Nordic countries. ASSIA, Sociological Abstracts, Social Services Abstracts. 2.9.2019

S8 (haku 3 S1 AND S3 AND S4) (100)

S7 (haku 2 S1 AND S2 AND S3 AND S4 (29)

S6 S1 AND S2 AND S3 AND S4 AND S5 (25)

S5 ("systematic review" or "scoping review" or meta-analysis or "randomized controlled trials as topic" or RCT or "randomized trial" or "randomised trial" or "randomised controlled" or "randomized controlled" or "randomized clinical" or "randomised clinical" or cohort* or cct or "controlled trial" or "controlled clinical" or follow-up or baseline or longitudinal or "before and after" or "before-after" or "parallel treatment" or "parallel group" or outcome* assessment or "parallel group" or "parallel treatment" or "clinical trial" or "implementation study" or "intervention study" or "treatment outcome" or "policy evaluation" or "evaluation study" or "comparative study") (409,057)

S4 TI (Finland* or finnish or scandinavia* or "nordic countries" or "nordic region" or swed* or denmark* or danish or norw* or iceland* or Faeroe* or Greenland*) OR AB(Finland* or finnish or scandinavia* or "nordic countries" or "nordic region" or swed* or denmark* or danish or norw* or iceland* or Faeroe* or Greenland*) OR SU(Finland* or finnish or scandinavia* or "nordic countries" or "nordic region" or swed* or denmark* or danish or norw* or iceland* or Faeroe* or Greenland*) (61,539)

S3 ("expert* by experience" or "health information" or "public information" or "information steering" or governance or "community policy" or "community program*" or "community intervention*" or "local policy" or "local policies" or "community program*" or "community intervention*" or "community-based intervention*" or "community-based program*" or "community health intervention*" or "community health program*" or "community health" or "community environment" or "multilevel intervention*" or "public health program*" or "health planning" or "health policy" or "policy planning" or "capacity building" or "built environment*" or "environmental design" or "environmental health" or "social environment" or "environment* planning" or "urban planning" or "city planning" or "rural planning" or "town planning" or "recreational facilities" or "sport* facilities" or "exercise facilities" or "school based" or "school health" or "school program*" or "school intervention*" or "school environment" or "school setting*" or curricul* or extracurric* or "day care" or kindergarten* or nursery or nurseries or "early childhood education" or "family center*" or "family centre*" or "youth centre*" or "youth center*" or outreach or "third sector" or volunteers or NGO* or "Non-governmental organi*" or church* or parish* or congregation or "public intervention*" or "public program" or "voluntary service*" or "community network*" or "voluntary organisation*" or "neighborhood association*" or "civic association*" or "voluntary organization*" or "social media" or "social marketing") (440,647)

S2 TI ( promot* or prevent* ) OR AB ( "health promot" or "disease prevention" or "public health prevent*" or "preventive medicine" or "preventive health" or "primary prevent*" or "primordial prevent*") OR SU (prevent* or promot* or "health promot" or "disease prevention" or "public health prevent*" or "preventive medicine" or "preventive health" or "primary prevent*" or "primordial prevent*" or “public health”) (19,597)

S1 TI("physical activity" or "physical fitness" or sports or "sporting activit*" or exercise or inactivity or "lack of exercise" or "low physical activity" or "lack of physical activity" or "minimal physical activity" or "insufficient physical activity" or "lack of exercise" or "little exercise" or "no exercise" or "physical* passiv*" or "inactive lifestyle" or sedentary or sitting or "stationary behav*" or "Stationary bout*" or "Stationary interruption*" or "Stationary break*" or "excessive stationary" or "stationary time" or "movement behav*" or "passive lifestyle" or "screen time") OR SU("physical activity" or "physical fitness" or sports or "sporting activit*" or exercise or inactivity or "lack of exercise" or "low physical activity" or "lack of physical activity" or "minimal physical activity" or "insufficient physical activity" or "lack of exercise" or "little exercise" or "no exercise" or "physical* passiv*" or "inactive lifestyle" or sedentary or sitting or "stationary behav*" or "Stationary bout*" or "Stationary interruption*" or "Stationary break*" or "excessive stationary" or "stationary time" or "movement behav*" or "passive lifestyle" or "screen time") (40,882)

ASSIA, Sociological abstracts, Social Services Abstracts (First round) 6.6.2019

TI("physical activity" or "physical fitness" or sports or "sporting activit*" or exercise or inactivity or "lack of exercise" or "low physical activity" or "lack of physical activity" or "minimal physical activity" or "insufficient physical activity" or "lack of exercise" or "little exercise" or "no exercise" or "physical* passiv*" or "inactive lifestyle" or sedentary or sitting or "stationary behav*" or "Stationary bout*" or "Stationary interruption*" or "Stationary break*" or "excessive stationary" or "stationary time" or "movement behav*" or "passive lifestyle") AND su(promot* OR prevent*) AND ("health education" OR "health information" OR "public information" OR "information steering" OR governance OR "community policy" OR "community program*" OR "community intervention*" OR "community-based intervention*" OR "community-based program*" OR "community health intervention*" OR "community health program*" OR "community health" OR "community environment" OR "multilevel intervention*" OR "public health program*" OR "health planning" OR "health policy" OR "built environment*" OR "environmental design" OR "environmental health" OR "social environment" OR "environment* planning" OR "urban planning" OR "city planning" OR "rural planning" OR "town planning" OR "recreational facilities" OR "sport* facilities" OR "exercise facilities" OR "school based" OR "school health" OR "school program*" OR "school intervention*" OR "school environment" OR "school setting*" OR curricul* OR "day care" OR kindergarten* OR nursery OR nurseries OR "early childhood education" OR "family center*" OR "family centre*" OR "youth centre*" OR "youth center*" OR outreach OR "third sector" OR NGO* OR "Non-governmental organi*" OR "public intervention*" OR "public program" OR "voluntary service*" OR "community network*" OR "voluntary organisation*" OR "neighborhood association*" OR "civic association*" OR "voluntary organization*" OR "social media" OR "social marketing") AND (Europ* OR Finland* OR finnish OR scandinavia* OR "nordic countries" OR "nordic region" OR swed* OR denmark* OR danish OR norw* OR iceland* OR scotland* OR scottish OR uk OR "united kingdom*" OR Canad* OR Holland* OR dutch OR Netherlands OR "New zealand*" OR Australia*) AND (("systematic review" OR "scoping review" OR meta-analysis OR "randomized controlled trials as topic" OR RCT OR "randomized trial" OR "randomised trial" OR "randomised controlled" OR "randomized controlled" OR "randomized clinical" OR "randomised clinical" OR cohort*) ) (146)
